# Identification of osteoblastic autophagy-related genes for predicting diagnostic markers in osteoarthritis

**DOI:** 10.1016/j.isci.2024.110130

**Published:** 2024-05-27

**Authors:** Rulong Cai, Qijun Jiang, Dongli Chen, Qi Feng, Xinzhi Liang, Zhaoming Ouyang, Weijian Liao, Rongkai Zhang, Hang Fang

**Affiliations:** 1 Department of Joint Surgery, Center for Orthopedic Surgery, The Third Affiliated Hospital, Southern Medical University, Guangzhou, 510630, China; 2 Academy of Orthopedics · Guangdong Province, Guangzhou, 510630, China; 3 Orthopedic Hospital of Guangdong Province, Guangzhou, 510630, China; 4 Guangdong Provincial Key Laboratory of Bone and Joint Degeneration Diseases, Guangzhou, 510630, China; 5 The Third School of Clinical Medicine, Southern Medical University, Guangzhou, 510515, China; 6 Department of Urology, The Third Affiliated Hospital, Southern Medical University, Guangzhou, 510630, China; 7 Department of Ultrasound, The University of Hong Kong-Shenzhen Hospital, Shenzhen, 518053, China

**Keywords:** Orthopedics, Human genetics, Bioinformatics

## Abstract

The development of osteoarthritis (OA) involves subchondral bone lesions, but the role of osteoblastic autophagy-related genes (ARGs) in osteoarthritis is unclear. Through integrated analysis of single-cell dataset, Bulk RNA dataset, and 367 ARGs extracted from GeneCards, 40 ARGs were found. By employing multiple machine learning algorithms and PPI networks, three key genes (DDIT3, JUN, and VEGFA) were identified. Then the RF model constructed from these genes indicated great potential as a diagnostic tool. Furthermore, the model’s effectiveness in predicting OA has been confirmed through external validation datasets. Moreover, the expression of ARGs was examined in osteoblasts subject to excessive mechanical stress, human and mouse tissues. Finally, the role of ARGs in OA was confirmed through co-culturing explants and osteoblasts. Thus, osteoblastic ARGs could be crucial in OA development, providing potential diagnostic and treatment strategies.

## Highlights

• Investigated the relationship between osteoblastic ARGs and OA
• Multiple machine learning approaches identified 3 key ARGs for OA diagnosis
• Experimental validation of ARGs *in vivo* and *vitro*

## Graphical abstract

**Figure:**
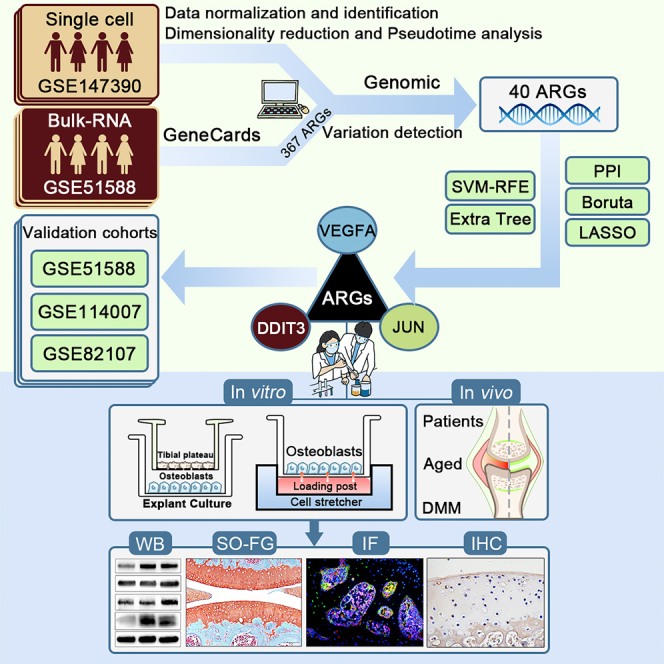

## Introduction

Osteoarthritis (OA) is the most prevalent debilitating joint disease, significantly impacting quality of life. It is estimated that by 2021, over 500 million people worldwide have been affected by OA.^1^ Although there are many ways to treat OA, none of them can achieve a radical cure.^2^ Prevention of OA seems to be the most effective means.^3^^,^^4^ In recent years, multiple studies have indicated that the primary causes of OA are aging, heredity, obesity, and mechanical stress.^5^ OA is a prevalent chronic degenerative joint condition, distinguished by articular cartilage degeneration, subchondral bone remodeling, meniscus degeneration, osteophyte formation, synovitis, and subpatellar fat pad. However, despite these well-recognized factors, the exact etiology of OA remains under investigation.^6^

Osteoblasts develop from neural crest progenitor cells and mesodermal cells, ultimately maturing into osteocytes. They have the ability to secrete various bioactive substances and to regulate and influence bone formation and remodeling.^7^^,^^8^ A growing body of research indicates that the development of OA can be influenced by the interaction between bone and cartilage.^9^ Recent evidence, alongside our research, confirms that abnormalities in the subchondral bone occur even before cartilage degeneration.^10^^,^^11^^,^^12^^,^^13^^,^^14^ The subchondral bone’s main role in early-stage OA development is to regulate bone resorption primarily through osteoclasts.^15^^,^^16^^,^^17^^,^^18^ In the late-stage, bone formation is mainly caused by overactive osteoblasts.^19^ However, during the early stage of OA, changes in the genes of osteoblasts may occur, causing dysfunction and bone remodeling, which is associated with OA development.^20^ Subchondral bone lesions primarily consist of sclerosis, osteophyte formation, and thinning of bone trabeculae. Moreover, osteophyte formation and subchondral bone sclerosis are mainly driven by osteoblasts.,^14^^,^^17^^,^^21^ Therefore, current researches focus on understanding the role of osteoblasts in the development of OA.

Autophagy is a vital intracellular protective process that serves to ensure cell survival and maintain cell homeostasis. This process involves the removal of long-lived, aggregated, and misfolded proteins, the renewal of damaged organelles, as well as the regulation of cell growth or senescence in normal physiological processes.^22^ Furthermore, autophagy is essential for cell differentiation, development, nutritional starvation, pathogen decomposition, and other vital biological processes.^23^ Autophagy plays a central role in maintaining cell morphology and function by serving as the key intracellular degradation process through lysosomes. Moreover, autophagy is also crucial for maintaining bone homeostasis.^24^

Recently, a growing number of studies have demonstrated the significant role of autophagy in osteoblasts, including its involvement in essential physiological processes such as bone development, remodeling, and metabolism.^25^ However, autophagy changes can disrupt osteoblast homeostasis, thereby causing a range of diseases.^19^ Recent studies have discovered that overactivation of the autophagy pathway can cause dysfunction and excessive proliferation of osteoblasts in the subchondral bone of OA patients.^19^ Numerous autophagy-related genes (ARG) and signal pathways, such as ATGs, mTOR, AMPK, ULK1, FoxO, ERK, and NF-κB, are crucial regulators in the onset and progression of OA.^26^^,^^27^ However, these findings are mainly concentrated in the field of chondrocyte research. The potential role and mechanism of osteoblast autophagy in OA remain unclear, and there has been no systematic summary and analysis of the ARGs in OA.

This study aimed to investigate the association between the autophagy function of subchondral osteoblasts and OA, as well as to identify the key ARGs influencing the osteoblast phenotype and their potential diagnostic significance. In this regard, bioinformatics methods were employed to identify differentially expressed genes (DEGs) and to predict the correlation and interaction of signal pathways. Additionally, the machine learning algorithms were utilized to identify key genes, and its reliability and robustness in diagnosing OA were evaluated by RF model. This was followed by the validation of the autophagy-related DEGs using overloading osteoblast and human OA tissue samples as well as mouse models of traumatic and age-related OA. In addition, the role of ARGs in OA was verified by co-culture of explants and osteoblasts. In all, the comprehensive understanding of the interplay between osteoblast autophagy and OA will potentially offer novel insights and directions for the diagnosis and targeted therapy of patients with OA.

## Results

### Single-cell landscape and extraction of feature genes

**Figure 1:**
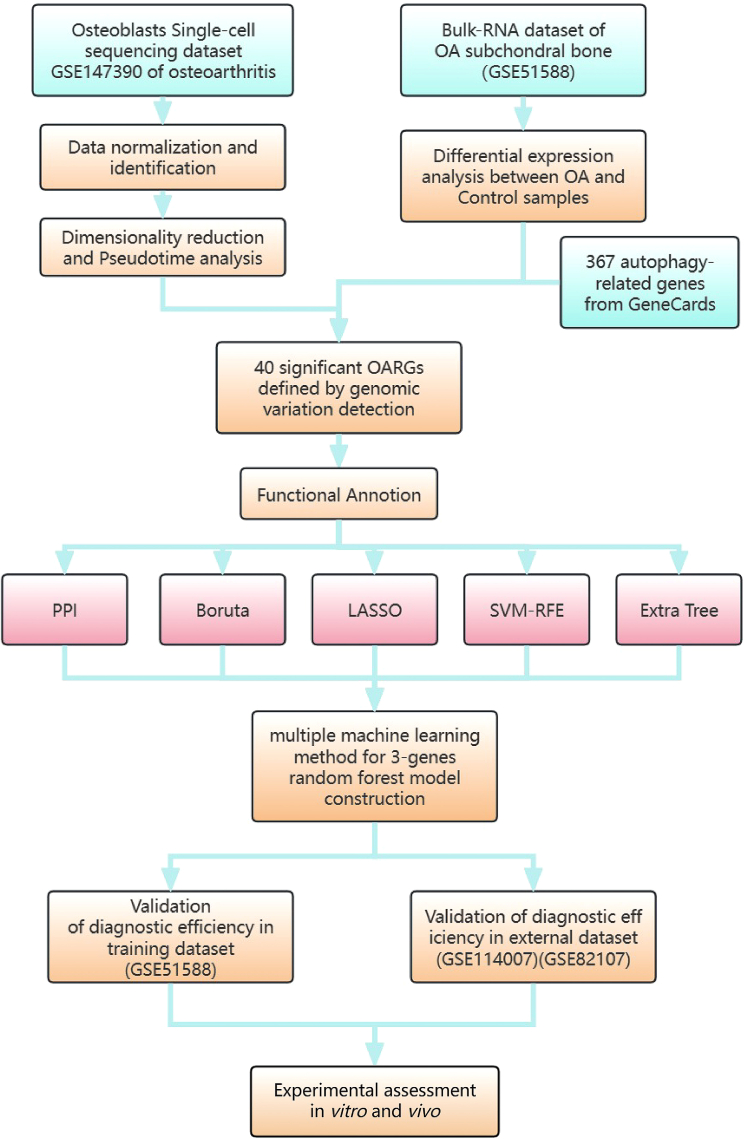
The work flowsheet of this study

**Figure 2:**
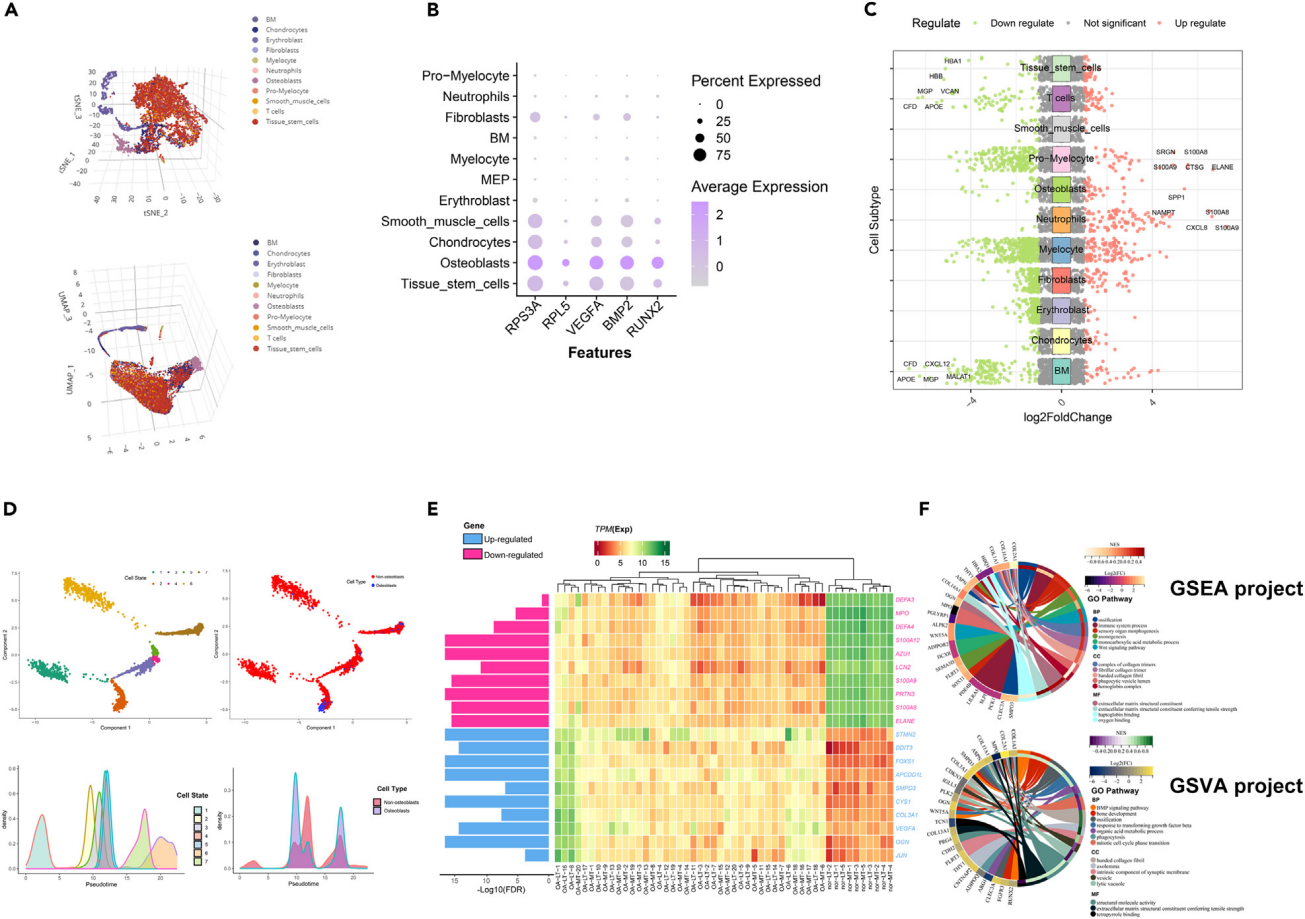
Investigation of single-cell dataset and transcriptome sequencing in OA (A) The cellular cluster distribution of Sc-RNA dataset GSE147390 using the T-SNE and U-MAP dimensionality reduction visualization methods. The osteoblast cells were identified by selected markers. (B) Highly variable genes expressed in osteoblasts cluster. (C) A volcano plot of significantly DEGs expression across all the cellular clusters in the GSE147390 dataset. (D) Pseudotime analysis atlas for seven developmental stages. The osteoblasts are further classified into pre-osteoblasts, mature osteoblasts, and a rare subset of osteoblasts. The red color represents identified osteogenic-related cells, while the green color indicates background cells. (E) The DEGs from the standardized RNA transcriptomic dataset GSE51588. The *p*-values represented as the negative logarithm. (F) The circle diagram of Gene Ontology (GO) pathways scores in GSE51588 with the GSEA and GSVA methods. FDR, false discovery rate; LogFC, log2(fold change); NES, normalized enrichment score.

The procedural steps of this study are depicted in Figure 1. To investigate the gene expression patterns of osteoblasts in OA, we downloaded the single-cell sequencing dataset GSE147390, which includes a femoral head sample from one OA patient, aiming to explore the specific expressed genes and lineages of osteoblasts in detail. After quality filtering of the expression matrix, the PCA-reduced single-cell samples were subjected to the SNN modularity optimization-based clustering algorithm (Figure S1), revealing distinct cell clustering patterns at resolutions ranging from 0.1 to 0.8 (Figure S2). The results demonstrated that the most reliable cell clustering occurred at a resolution of 0.4 (where clusters 0 and 4 belong to the same class). Through cell identification, we identified 12 cell types and determined the cluster most similar to osteoblasts based on self-collected marker genes^28^ (Figure S3). UMAP clustering and TSNE clustering are presented on Figure 2A. Based on cell annotation, we identified specific upregulated and downregulated genes for each cluster using the volcano plot (Figure 2C). Additionally, we highlighted highly variable genes, such as RPL5, VEGFA, and BMP2 (Figure 2B), which ranked among the top in the osteoblast cluster, via dot plots. Furthermore, we employed pseudotime analysis to unveil the different developmental states of OA cells, defining 1 to 7 cell state using the Monocle package and presenting them separately. Within the developmental timeline, osteoblasts were divided into three different stages, with the majority of cells located in stage 2 of the overall developmental tree (Figure 2D). This suggests that osteoblasts labeled using the cell markers collected in this study can be classified into three subgroups: pre-osteoblasts, mature osteoblasts, and an undetermined rare osteoblast subpopulation. These findings align with the expected outcomes.

### Identification of osteoblastic autophagy-related genes

After |Log(2 + X)| standardization of the GSE51588 cohort, we employed the R packages “limma”, “DESeq”, and “edgeR” to analyze the significant differential genes between OA patients and the normal samples. Combining all the results, we obtained a total of 211 significant differential genes, including 136 upregulated genes and 75 downregulated genes. To visualize the distribution of selected DEGs in different cohorts and groups, we employed a heatmap (Figure 2E). Subsequently, we utilized the GSEA and GSVA method to assess the enrichment of significant proteins within GO terms, aiming to elucidate the signaling pathways and biological functions of DEGs (Figure 2F). Observation findings indicate that monocarboxylic acid metabolic process and immune system process related pathways are inhibited in biological processes (BPs), while pathways associated with positive regulation of bone development and ossification are activated in OA. This suggests a regulatory association between DEGs and the subchondral bone growth and development of OA. In the cellular component (CC) module, biological functions enriched with DEGs revealed an upregulation of fibrillar collagen trimer and banded collagen fibril in subchondral bone of the joint. Previous studies have found that where the compressive stress of OA is obvious, subchondral bone sclerosis is significant, and the expression of Col I and the thickness of Col I fibers are also increased.^29^ This indicates a close association between DEGs and cartilage development. In terms of molecular function (MF), we also observed a decrease in activities of small molecule binding functions, such as tubulin binding^30^^,^^31^ and pyrrole binding^32^ in the OA group, while extracellular matrix structural components conferring tensile strength and structural molecule activity were active. This indicates the involvement of DEGs in the production of subchondral bone extracellular matrix and their association with the growth and development of osteoblasts, which is as previously reported.^33^

**Figure 3:**
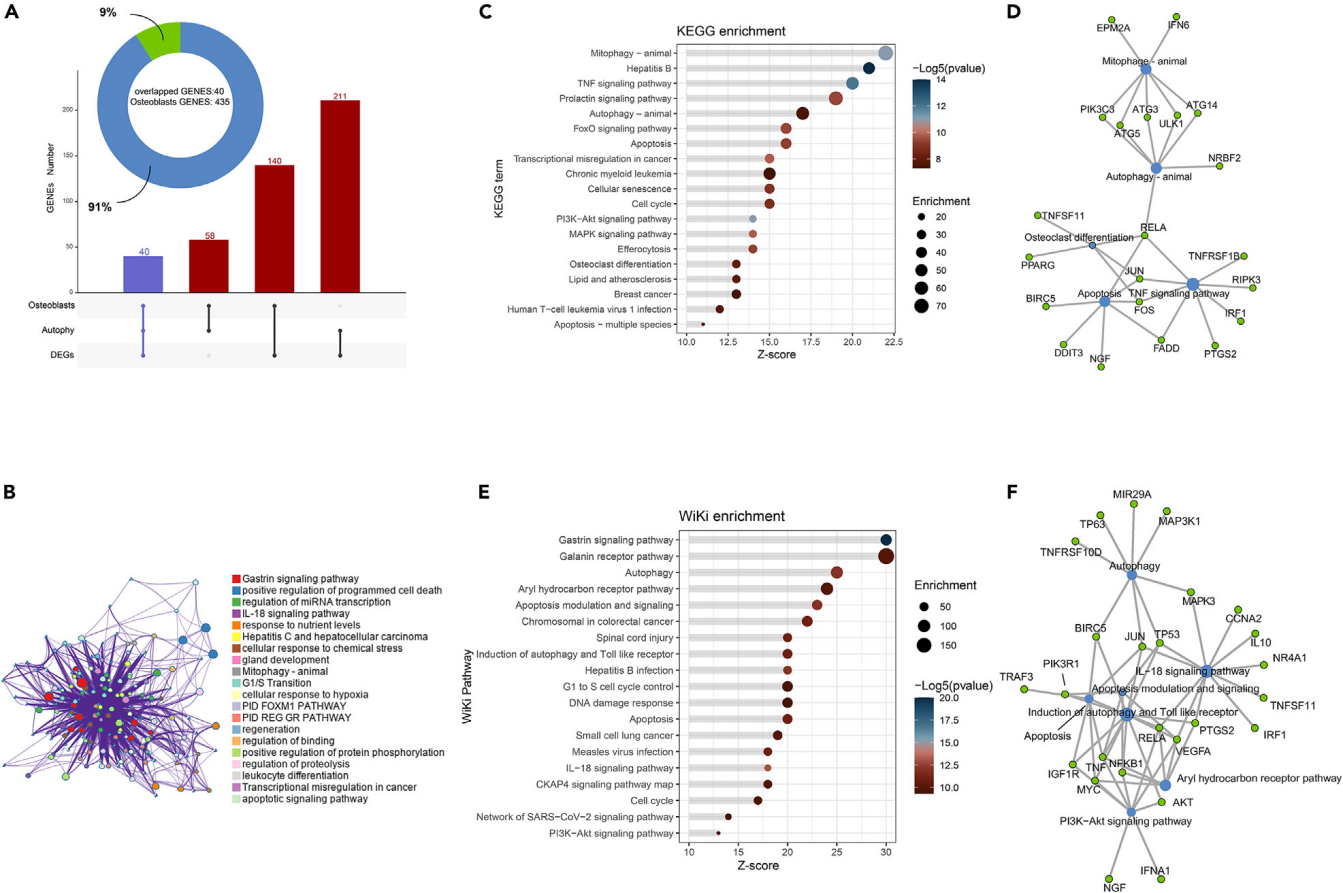
The biofunctional annotation of the osteoblastic ARGs (A) Identification of 40 overlapped osteoblasts autophagy-related genes. (B) The pathway enrichment analyses via the Metascape database. (C–F) Dotplot of KEGG (C) and WIKI (E) pathway annotation associated with 40 ARGs, and the internal protein interaction network (D and F) of partially substantial pathways. FDR, false discovery rate; LogFC, log2(fold change).

To further identify effective targets for the diagnosis of OA, we introduced ARGs and combined bulk RNA sequencing with single-cell data to screen 40 osteoblast ARGs (Figure 3A). To investigate the potential functional annotations of these 40 genes, we uploaded them to the Metascape database and performed KEGG and Wiki pathway enrichment analyses. The enriched pathways, their interactions, and importance are shown as follow (Figure 3B). The analysis reveals that the associated genes are involved in KEGG pathways such as positive regulation of Mitophagy-animal, TNF signaling pathway, Autophagy-animal and PI3K-AKT signaling pathway, which are crucial for joint subchondral bone development and remodeling. (Figure 3C) Meanwhile, the Wiki pathways mainly focus on apoptosis modulation and signaling, Autophagy, Induction of autophagy and Toll like receptor, and so on (Figure 3E). In addition, the co-expression network graph illustrates the interconnectedness of core genes in these key pathways (Figures 3D and 3F). It is evident that co-expression of core genes significantly overlaps to a large extent.

### Machine learning algorithms and PPI of genes identification

**Figure 4:**
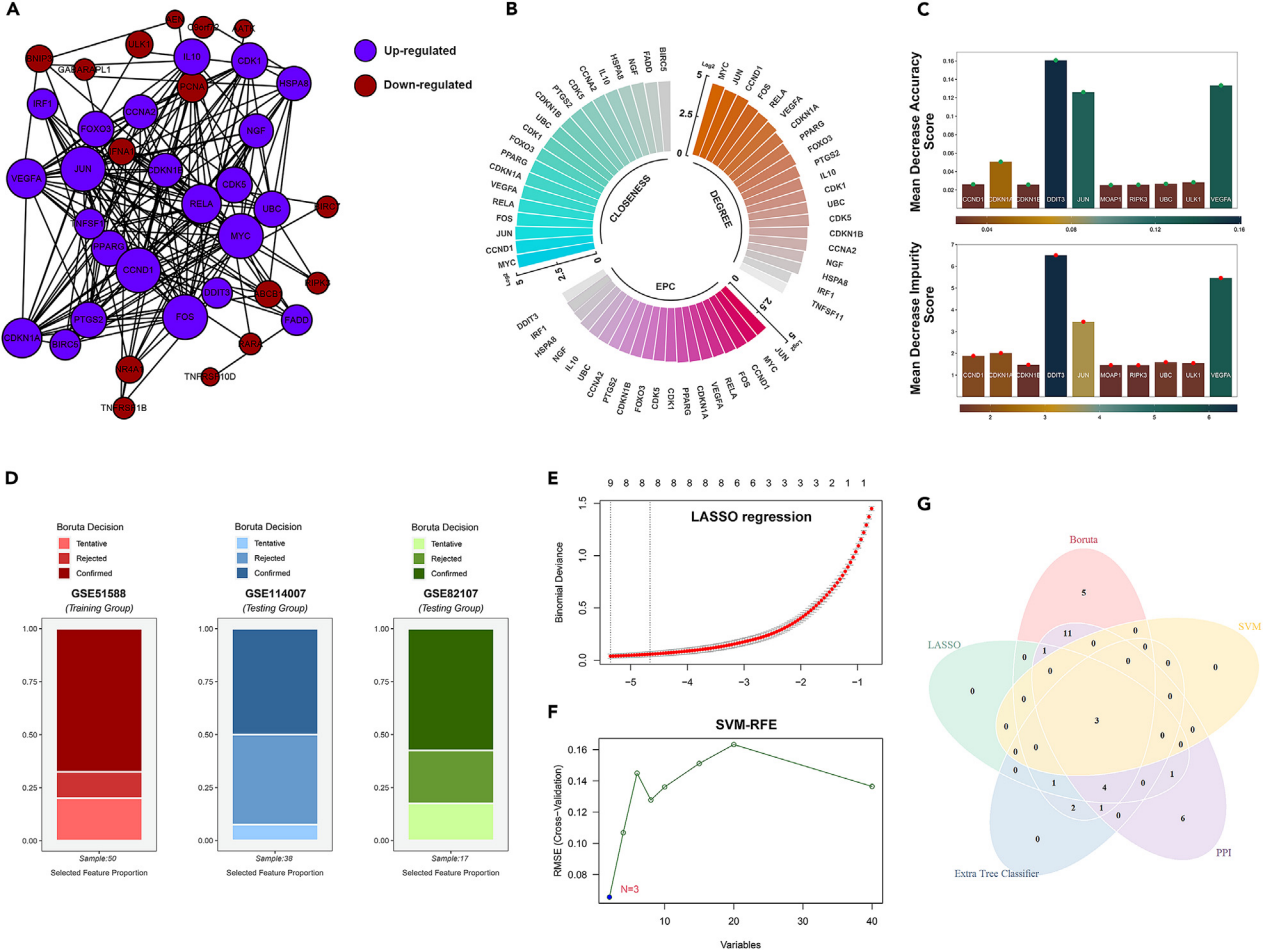
Incorporating various machine learning techniques and protein-protein interaction (PPI) networks into the comprehensive screening of osteoblastic ARGs (A) The interplay relationships within the PPI network of relevant genes. (B) Sorting of the top 20 crucial genes for DEGREE, CLOSENESS, and EPC algorithm from Cytoscape software. (C) Mean decrease Impurity score and mean decrease Accuracy coefficient of each marker in Extra Tree Classifier model. (D) The proportion of Boruta algorithm decision across training dataset and testing dataset. (E) The optimal number of features that ensures the best predictive performance through lasso regression analysis. (F) The SVM-RFE method further compresses the variable features. (G) The most significant features DDIT3, JUN and VEGFA are jointly determined by the PPI network analysis and multiple machine learning algorithms.

We clarify the interplay relationships and co-expression disparities of the selected 40 proteins, the String database, widely regarded as the most utilized tool, was employed to construct the interaction network and illustrate the co-expression relationships among core proteins (Figure 4A). Evaluative algorithms from the Cytoscape plug-in unit were utilized to further filter the key genes in the network. The condensed metrics comprised 15 downregulated factors and 22 upregulated factors. (Figure 4B) Subsequently, the Boruta machine learning model (Figure 4D) was employed to assess the most significant features contributing to the development of OA, out of which 9 were further determined as candidate feature parameters for the predictive model through Lasso regression algorithm (Figure 4E). To augment the conciseness and robustness of the predictive model, additional machine learning feature selection methods were concurrently applied. The SVM-RFE algorithm identified 3 variables (Figure 4F), whereas the extra tree classifier incorporated 11 variables (Figure 4C), all of which exhibited statistical significance with the outcomes. Ultimately, through the joint determination of machine learning algorithms and protein-protein interaction (PPI) network analysis, three pivotal features were identified and included in the arthritis diagnostic model: AP-1 transcription factor subunit (JUN), DNA-damage inducible transcript 3 (DDIT3), and vascular endothelial growth factor A (VEGFA) (Figure 4G).

### Validating the osteoblastic ARGs model of OA

**Figure 5:**
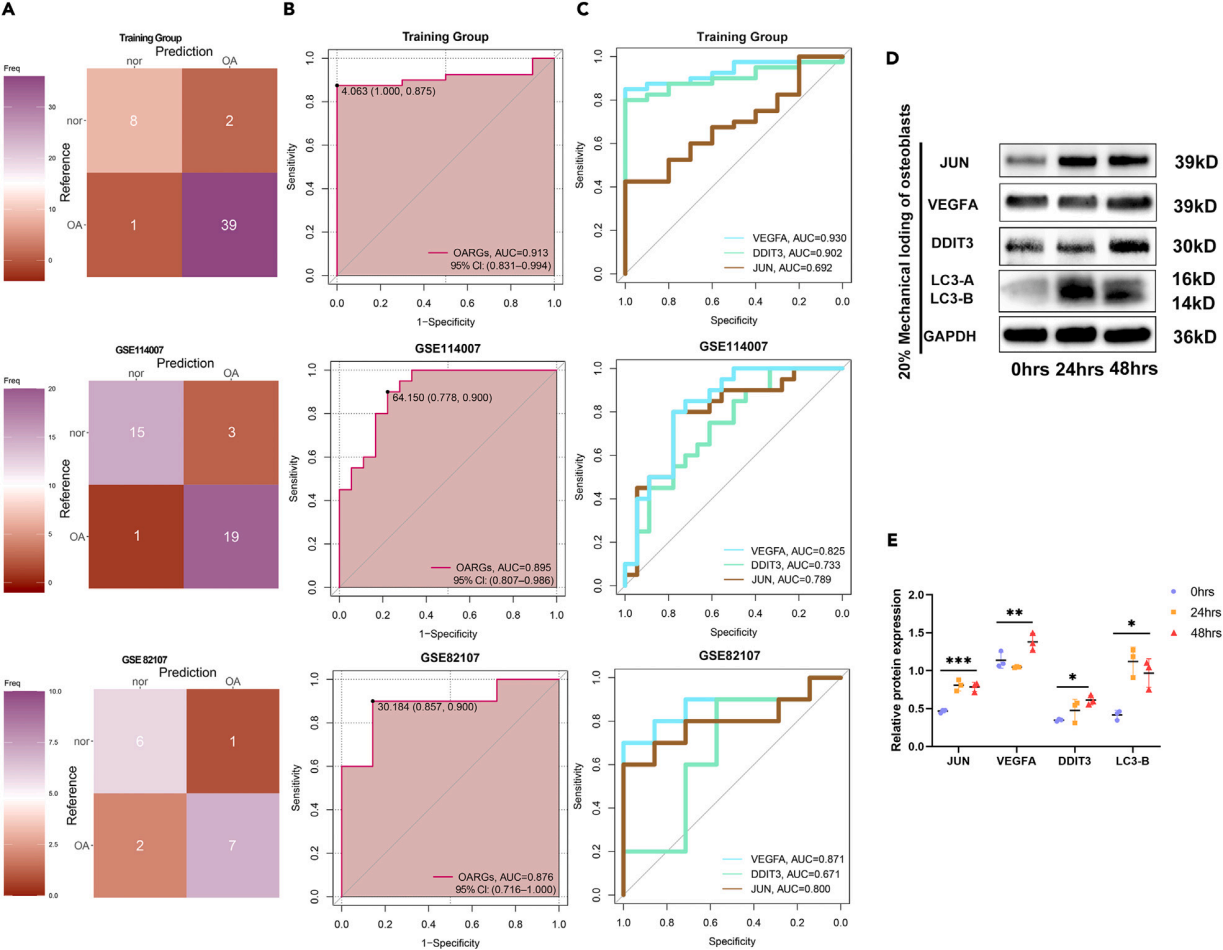
The assessment of diagnostic efficiency within the GSE51588, GSE114007 and GSE82107 dataset for the random forest model prediction (A) The confusion matrix displays the predictive efficacy of the osteoblastic ARGs model across Training (GSE51588) dataset, GSE114007 and GSE82107 dataset. Sample of True negative (top left), true positive (bottom right), false negative(bottom left) and false positive(top right) have been shown. Red represents less number; purple represents more. (B) The ROC curve of 3-gene random forest model to training group and another 2 external validation groups. (C) The ROC analyses exhibit DDIT3, VEGFA, and JUN respective OA diagnostic performance within training and validation datasets. (D) Western blot analysis the expression of JUN, VEGFA, DDIT3 in 20% mechanical loading of osteoblasts. (E) Quantification of the expression of JUN, VEGFA, DDIT3 in 20% mechanical loading of osteoblasts. (∗*p* < 0.05; ∗∗*p* < 0.01; ∗∗∗*p* < 0.001; ns, *p* > 0.05).

We developed an OA diagnostic model based on three osteoblast ARGs using the random forest (RF) method with the SVM package. The model parameters in the risk score are as follows: ARGs = 0.235 ∗ JUN +0.85 ∗ VEGFA +0.478 ∗ DDIT3. Confusion matrices constructed using the R package “likert” for the training dataset and validation datasets are presented in Figure 5A, respectively. The RF risk model exhibited effectiveness and stability in differentiating OA samples from control cases in the training dataset GSE51588, achieving an AUC of 0.913 (95%CI: 0.831–0.994, Figure 5B), as well as in the GSE114007 and GSE82107 datasets with an AUC of 0.895 (95%CI: 0.807–0.986) and 0.876 (95%CI: 0.716–1.000). Furthermore, we constructed ROC curves to assess the diagnostic capability of the selected biomarkers in distinguishing OA and control samples. JUN (AUC = 0.692), VEGFA (AUC = 0.93), and DDIT3 (AUC = 0.902) exhibited high AUC values (Figure 5C). These findings were subsequently validated in two additional cohorts. In the GSE114007 cohort, all three key indicators displayed good diagnostic performance, with AUC values for VEGFA = 0.825, JUN = 0.789, and DDIT3 = 0.733. Similarly, the GSE82107 cohort exhibited favorable diagnostic efficacy for VEGFA, JUN, and DDIT3, with AUC values of 0.871, 0.800, and 0.671, respectively. Moreover, our *in vitro* experiments involved subjecting osteoblasts to excessive mechanical loading to simulate an arthritis model. In doing so, we observed varying degrees of increase in the expression of these three proteins at the protein level (Figures 5D and 5E), highlighting a discernible relationship between the expression of these proteins and mechanical loading in osteoblasts. In addition, we analyzed the expression of ARGs in each database. The results revealed that DDIT3, JUN and VEGFA exhibited significant upregulation in subchondral bone of OA patients (GSE51588) (Figure S4A), while these three markers were downregulated in OA cartilage (GSE114007) (Figure S4B) and synovium (GSE82107) (Figure S4C), which was consistent with previous research reports. Many studies have found that autophagy of cartilage and synovium is primarily inhibited in OA.^34^^,^^35^^,^^36^^,^^37^ However, this does not affect the diagnostic efficiency of the parameters of the whole model. Hence, these findings strongly suggest an increase in autophagy in the subchondral bone of OA, aligning with our expectations and emphasizing the high predictive reliability of these three feature genes in relation to OA.

### Osteoblastic autophagy-related genes were verified to be upregulated in mouse OA model

**Figure 6:**
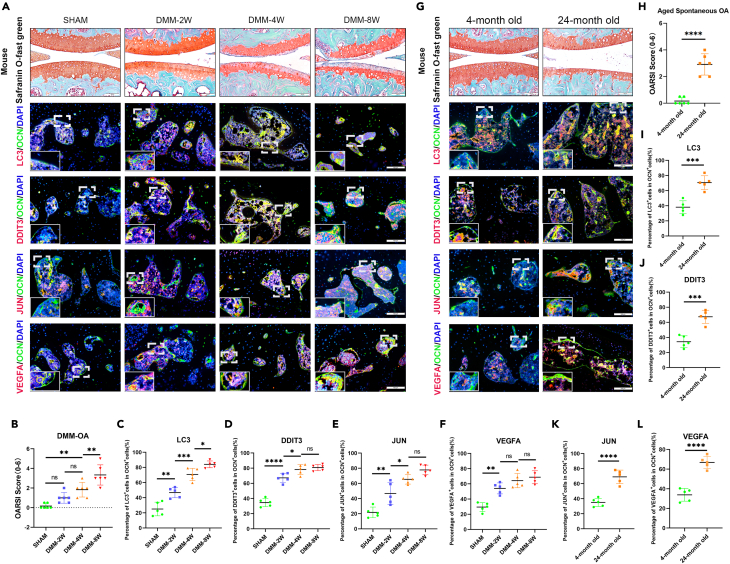
Osteoblastic autophagy-related genes were upregulated in mouse OA model (A) Representative images of safranin O/fast green staining (top) and immunofluorescence of LC3 and OCN, DDIT3 and OCN, JUN and OCN, VEGFA and OCN (middle and bottom) co-immunostaining in the tibial subchondral bone of controls and DMM mice. Scale bar = 100 μm. (B) Quantitative analysis of the OARSI score of controls and DMM mice. *n* = 5 per group. (C–F) Quantitative analysis of the percentage of LC3^+^, DDIT3^+^, JUN^+^, and VEGFA^+^ cells in OCN^+^ cells of controls and DMM mice. *n* = 5 per group. (G) Representative images of safranin O/fast green staining (top) and immunofluorescence of LC3 and OCN, DDIT3 and OCN, JUN and OCN, VEGFA and OCN (middle and bottom) co-immunostaining in the tibial subchondral bone of mice aged 4 and 24 months. Scale bar = 100 μm. (H) Quantitative analysis of the OARSI score of mice aged 4 and 24 months. *n* = 6 per group. Scale bar = 100 μm. (I–L) Quantitative analysis of the percentage of LC3^+^, DDIT3^+^, JUN^+^, VEGFA^+^ cells in OCN^+^ cells of mice aged 4 and 24 months. *n* = 5 per group. (∗*p* < 0.05; ∗∗*p* < 0.01; ∗∗∗*p* < 0.001; ∗∗∗∗*p* < 0.0001; ns, *p* > 0.05).

To induce the model of traumatic OA and age-related OA, we used DMM operation and aged mice. For DMM-OA mice, all knee joint specimens were collected at 2, 4, and 8 weeks after DMM operation. For aged mice, we collected all knee joint specimens aged 4 months and 24 months. Then, we observed the decrease of proteoglycan and rough surface in articular cartilage of the OA group (Figures 6A and 6G) and the corresponding OARSI score of each group (Figures 6B and 6H) was evaluated by Safranin O-Fast Green staining, which showed that the cartilage matrix degeneration of OA model mice was more obvious than that of the control group. It is worth noting that the OA model induced by DMM surgery showed cartilage degeneration at 2 weeks post-operation. Furthermore, immunofluorescence staining for subchondral osteoblasts and autophagy was performed in each group to elucidate their relationship. Double immunofluorescence was used to observe the co-expression of ARGs and the osteoblast-specific marker OCN in OA samples. As anticipated, LC3 (Figures 6A, 6C, 6G, and 6I), DDIT3 (Figures 6A, 6D, 6G, and 6J), JUN (Figures 6A, 6E, 6G, and 6K), and VEGFA (Figures 6A, 6F, 6G, and 6L), were strongly co-located with osteoblast marker OCN.

### Osteoblastic autophagy-related genes were verified to be upregulated in OA patients

**Figure 7:**
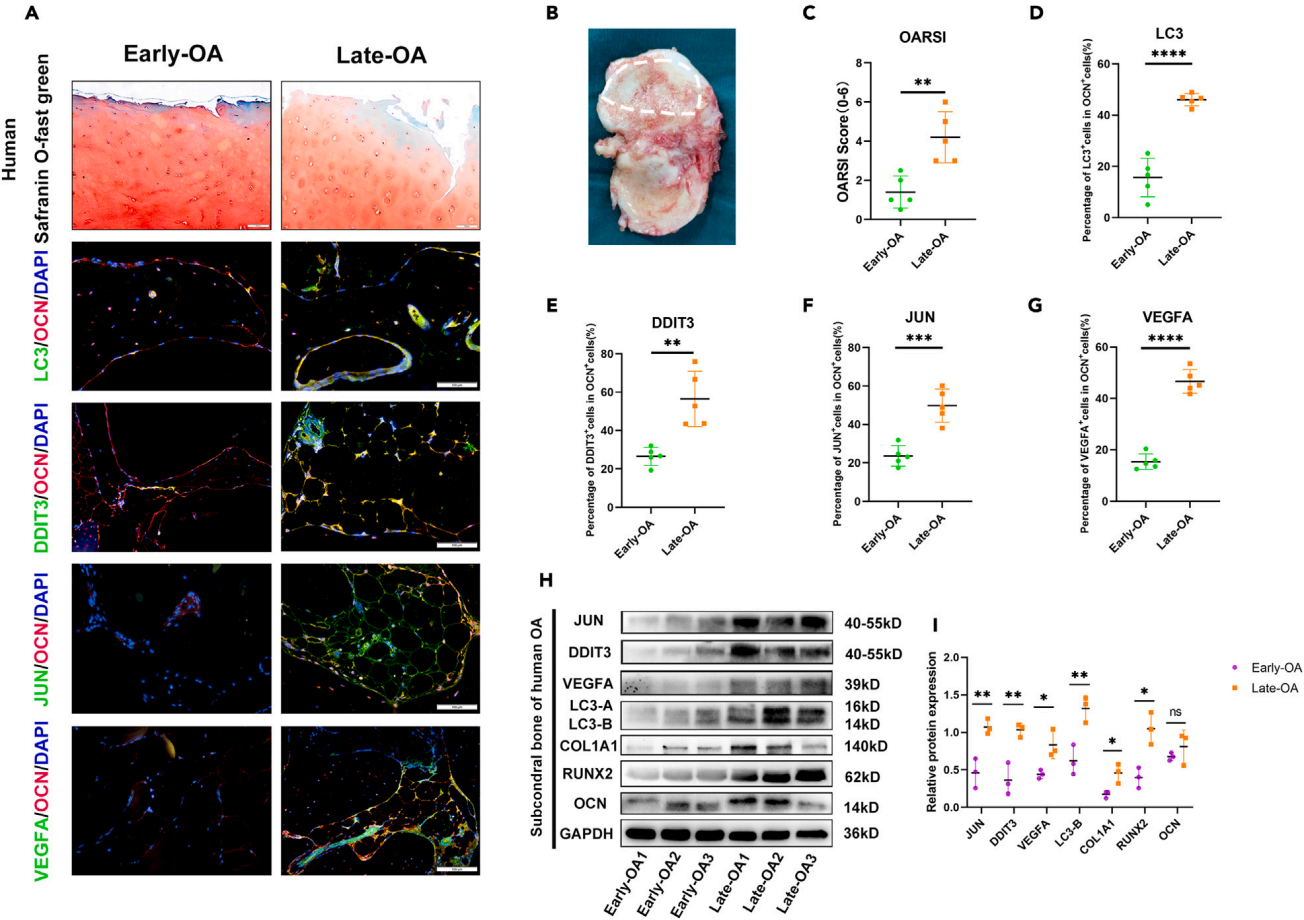
Osteoblastic autophagy-related genes were upregulated in OA patients (A) Representative images of safranin O/fast green staining (top) and immunofluorescence of LC3 and OCN, DDIT3 and OCN, JUN and OCN, VEGFA and OCN (middle and bottom) co-immunostaining in the tibial subchondral bone of OA patients. Scale bar = 100 μm. (B) Schematic diagram of tibial plateau tissue: in dotted line is the medial, corresponding to the Late-OA(OA group), and the opposite side is the lateral side which as the Early-OA(control group). (C) Quantitative analysis of the OARSI score of early-OA and late-OA. *n* = 5 per group. (D, E, F and G) Quantitative analysis of the percentage of LC3^+^, DDIT3^+^, JUN^+^, and VEGFA^+^ cells in OCN^+^ cells of early-OA and late-OA. *n* = 5 per group. (H) Western blot analysis the expression of JUN, DDIT3, VEGFA, LC3, COL1A1, RUNX2, and OCN in knee subchondral bone of OA. (I) Quantification of the expression of JUN, DDIT3, VEGFA, LC3, COL1A1, RUNX2, and OCN in knee subchondral bone of OA. (∗*p* < 0.05; ∗∗*p* < 0.01; ∗∗∗*p* < 0.001; ∗∗∗∗*p* < 0.0001; ns, *p* > 0.05).

To verify the expression of ARGs in subchondral osteoblasts in OA patients, we collected the tibial plateau of patients who were clinically diagnosed as OA and underwent total knee arthroplasty. We define the heavily worn side of the tibial plateau, that is, the medial side is the late OA, and the opposite side is the early OA (Figure 7B). Safrin-*O*-Fast green staining demonstrated that the cartilage surface of early OA was smoother and the loss of cartilage matrix was lighter. On the other hand, the late OA showed obvious defects on the cartilage surface and severe degradation of cartilage matrix (Figures 7A and 7C), which suggested our definition of OA tibial plateau is reasonable. Then, we used the immunofluorescence co-localization of LC3, DDIT3, JUN, and VEGFA with osteoblast-specific marker OCN, and found that they showed strong co-localization in the late OA group (Figures 7A and 7D‒7G). Furthermore, we conducted further *in vivo* evaluation of the expression levels of feature genes in the knee subchondral bone of human OA samples. Our Western blot results revealed a more pronounced activation of osteoblasts in the subchondral bone of late-stage OA. Concurrently, we observed upregulation in the expression of LC3, JUN, DDIT3, and VEGFA genes in late-stage OA osteoblasts, compared with early-stage OA (Figures 7H and 7I). As predicted, autophagy of OA subchondral osteoblasts is activated, and the predicted three related genes may be strong evidence for OA diagnosis.

### ARGs indirectly affect cartilage metabolism by regulating osteoblast activity

**Figure 8:**
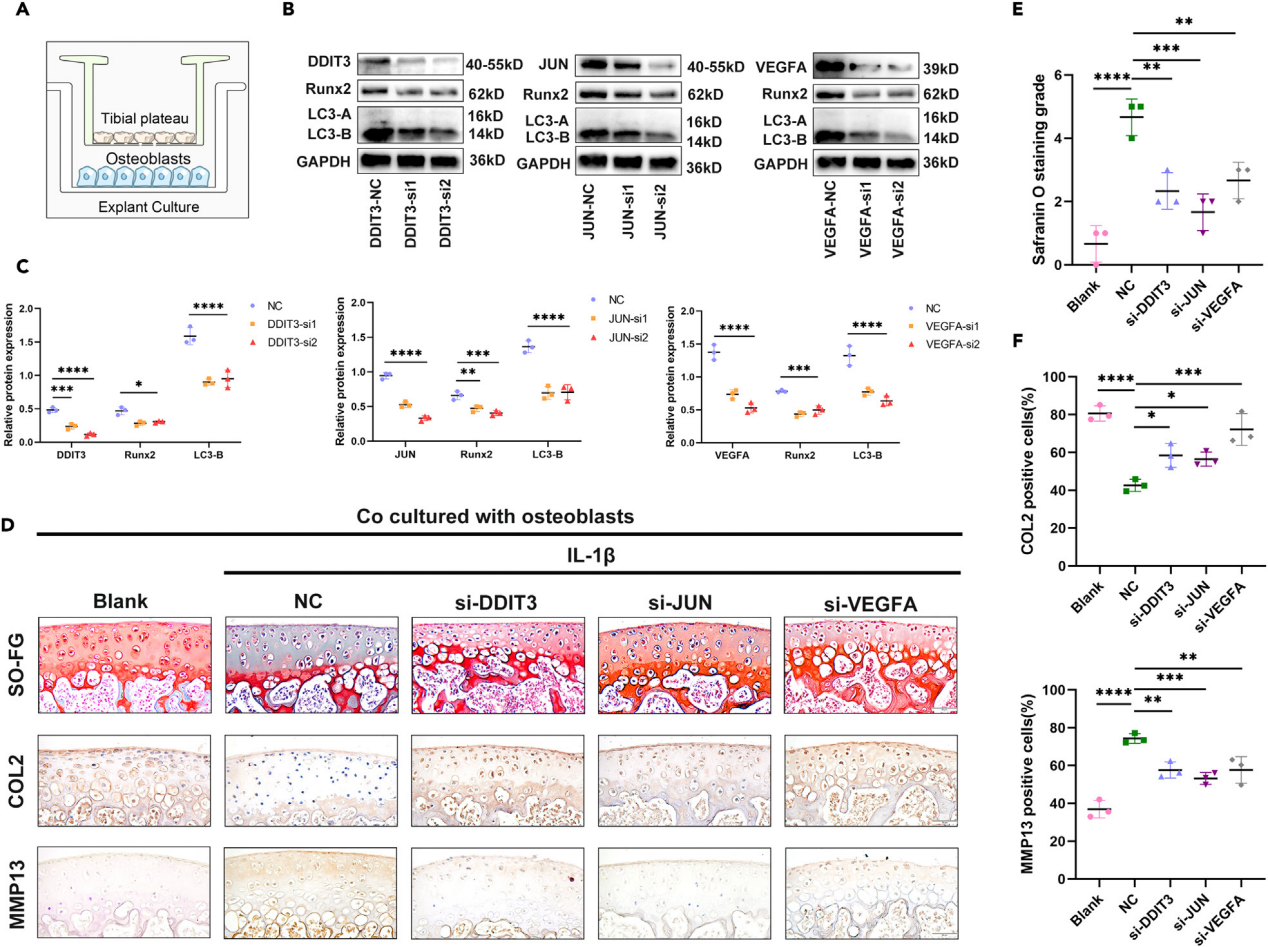
Osteoblastic ARGs indirectly affect cartilage metabolism through the regulation of osteoblast activity (A) Schematic diagram of co-culture of osteoblasts and tibial plateau cartilage explants. (B) Western blot analysis the expression of DDIT3, JUN, VEGFA, Runx2, and LC3 in osteoblasts transfected with siRNA. (C) Quantification of the expression of DDIT3, JUN, VEGFA, Runx2, and LC3 in osteoblasts transfected with siRNA. *n* = 3 per group. (D) Representative images of Safranin O-Fast Green staining (top) and immunohistochemistry (IHC) of COL2 and MMP13 (middle and bottom) in the mouse tibial plateau cartilage explants co-cultured with osteoblasts. Scale bar = 50 μm. (E) Quantitative analysis of Safranin O staining and grade of mouse tibial plateaus cartilage explants co-cultured with osteoblasts. *n* = 3 per group. (F) Quantitative analysis of COL2 and MMP13 in the mouse tibial plateau cartilage explants co-cultured with osteoblasts. *n* = 3 per group. (∗*p* < 0.05; ∗∗*p* < 0.01; ∗∗∗*p* < 0.001; ∗∗∗∗*p* < 0.0001; ns, *p* > 0.05).

In our previous experiments, it was confirmed that the expression of ARGs is upregulated in osteoblasts of OA. Herein, we aimed to assess the effect of ARGs on the cartilage metabolism of OA. To achieve this, we conducted siRNA transfections to knock down the DDIT3, JUN, and VEGFA genes in osteoblasts. Simultaneously, tibial plateau cartilage explants were obtained from 3-week-old mice. Subsequently, osteoblasts with reduced ARGs levels were co-cultured with IL-1β-stimulated cartilage explants *in vitro* for a period of 3 days to observe the indirect effect of ARGs on cartilage metabolism. The experimental setup for this co-culture model is depicted in Figure 8A. Our findings demonstrated successful knockdown of ARGs in the osteoblasts, leading to decreased expression levels of the LC3 and reduced activity index of Runx2 in the osteoblasts (Figures 8B and 8C). These results indicate that ARGs in osteoblasts can regulate osteoblast activity. Moreover, Safranin O-Fast Green staining revealed the obvious cartilage degradation of IL-1β-stimulated cartilage explant groups than that of blank group and a reduction in cartilage degradation in the explants following osteoblastic ARGs knockdown (Figures 8D and 8E). Additionally, immunohistochemistry (IHC) results of cartilage metabolism markers such as COL2 and MMP13 indicated enhanced cartilage anabolism and delayed degradation in the ARGs knockdown groups (Figures 8D and 8F). In conclusion, our study suggests that osteoblastic ARGs can indirectly influence cartilage metabolism by modulating osteoblast activity, ultimately playing a role in the development of OA.

## Discussion

OA is a degenerative disease of the whole joint. Subchondral bone remodeling is an important pathological change, but its exact pathological mechanism is not clear. It has been found that the abnormal activation of osteoblasts in subchondral bone mediates the progression of OA, and the inhibition of osteoblasts may change bone formation and reduce the degeneration of articular cartilage.^38^ Multiple studies have demonstrated that ARGs and pathways regulate the differentiation and function of osteoblasts. The activation of autophagy can increase the activity of osteoblasts, stimulate osteoblast-mediated bone formation and accelerate bone turnover. However, due to the complex pathogenesis of OA, there is no effective diagnostic marker at present. High-throughput sequencing technology can enhance the exploration of the potential mechanisms behind the occurrence and development of OA, facilitating the discovery of new and effective diagnostic biomarkers. Therefore, studying the regulation of osteoblast autophagy holds the potential to offer new insights into the specific mechanisms underlying OA, and may also contribute to advancements in its diagnosis and treatment. Therefore, employing bioinformatics to identify biomarkers and conducting subsequent biological experiments could represent a promising approach for investigating OA.

In analyzing the single-cell dataset GSE147390 from the GEO database, OA osteoblasts have been categorized into three subtypes: pre-mature osteoblasts, mature osteoblasts, and rare osteoblasts. Additionally, we utilized the OA GSE51588 database to conduct Gene Ontology (GO) enrichment analysis of significant proteins. The findings revealed the activation of pathways associated with positive regulation of bone development and osteogenesis in OA. Furthermore, upregulation of the fibrillar collagen trimer and banded collagen fibrils in subchondral bone was observed, alongside decreased activities in microtubule binding and pyrrole binding. Moreover, structural components of the extracellular matrix correlated with tensile strength and structural molecular activity showed increased activity. These results suggest that DEGs not only contribute to the remodeling of OA subchondral bone, but also participate in cartilage degradation and the production of extracellular matrix within the subchondral bone. Combining GSE147390 with GSE51588, the potential autophagy genes in osteoblasts were analyzed by bioinformatics method. The identification of autophagy genes was conducted through differential gene analysis in multiple transcripts group data, resulting in the identification of 40 differential genes by integrating single-cell data. It means that elucidating the key genes associated with this process holds promise for informing strategies aimed at the diagnosis and treatment of OA. The KEGG and WiKi pathway analyses showed that the autophagy-related DEGs were primarily linked to mitophagy and autophagy, as well as molecular pathways related to autophagy, such as FoxO and MAPK signaling pathways. At present, osteoblasts and osteoclasts are the main cells to promote bone metabolism and maintain the balance of bone metabolism.^39^ However, autophagy regulates osteogenesis and osteoclast activity, which involves a variety of bone metabolic disorders, including OA and osteoporosis.^40^^,^^41^^,^^42^ Our findings are basically consistent with the above research. Nevertheless, the role of these genes in OA is only emphasized by bioinformatics, and the genes that can really become biological diagnostic markers need further bioinformatics analysis.

To obtain more effective diagnostic markers, we constructed a PPI network to illustrate the co-expression relationship of these 40 genes. Evaluative algorithms from the Cytoscape plug-in unit screened 15 upregulated factors and 22 downregulated factors. Then, using the machine learning models including Boruta, Lasso, SVM and Extra Trees Classifier, combined with PPI network analysis, we finally obtained three genes with the most diagnostic significance of OA: DDIT3, VEGFA, JUN.

DDIT3 is a member of the CCAAT/enhancer binding protein (C/EBP) family of transcription factors, also referred to as C/EBP homologous protein, which participated in gene expression regulation.^43^^,^^44^ Furthermore, DDIT3 is crucial in mediating endoplasmic reticulum (ER) stress-mediated autophagy, apoptosis, energy metabolism, and cell differentiation. Besides, DDIT3 is related to regulating bone metabolism and dynamic balance.^45^ However, its role in regulating osteogenic differentiation is still controversial, as some studies show it promotes osteogenesis while others indicate it inhibits osteogenesis.^46^^,^^47^ In addition, DDIT3 also inhibits the mineralization of cementoblast.^48^ ER stress downstream molecule, DDIT3, can impact cartilage development and metabolism.^49^ However, the role and pathogenesis of DDIT3 in OA are still unclear, and further research is needed. VEGFA (vascular endothelial growth factor A), also known as VEGFA165, is one of the structure-related factors of the VEGF family and mainly activates ligands VEGFR1 and VEGFR2.^50^ VEGFA is a pro-angiogenic factor, which plays a key role in a variety of biological processes, including angiogenesis, cell proliferation and survival, and pain transmission.^51^ Additionally, VEGFA is widely expressed in bone tissue and is associated with osteoblasts. It activates signal transduction pathways by binding to receptors, ultimately impacting the differentiation, bone matrix synthesis, and proliferation of osteoblasts.^52^^,^^53^^,^^54^^,^^55^^,^^56^ We predicted and verified that VEGFA is upregulated in osteoblasts of OA, indicating that VEGFA is also involved in the activity and function of osteoblasts in the occurrence and development of OA. Several studies have demonstrated that VEGFA promotes the development of OA by inducing subchondral blood vessel formation, articular cartilage degeneration, and inflammatory response enhancement.^51^^,^^57^^,^^58^ JUN(AP-1 transcription factor subunit), whose transcription level increased in varying degrees in the process of osteoblast proliferation and differentiation.^59^ It is reported that AP-1 is involved in osteoclast CTSK production mediated by RANKL stimulation.^60^ In the aspect of cartilage, it is highlighted that the JNK-JUN-NCOA4 axis promotes ferroptosis of chondrocytes, leading to accelerated pathogenesis of OA via ferritinophagy.^61^ Besides, previous studies have found that the expression of c-fos and c-jun in synovial tissue of rheumatoid arthritis is likely to be caused by persistent inflammatory stimulation, which mediates the progression of RA disease.^62^ It was reported that the related proteins such as c-Jun, c-Fos, *p*-JNK in JNK-AP1 signal pathway are increased in osteosarcoma, suggesting that they may participate in the malignant transformation of osteoblasts in human osteosarcoma.^63^ Overall, these three genes are significant in osteoblasts and OA and could be valuable for diagnosing and treating OA.

To evaluate the diagnostic effectiveness of these ARGs in OA, we used RF model to analyze the GSE51588, GSE114007, and GSE82107 datasets, which finally demonstrated favorable diagnostic efficacy for VEGFA, JUN, and DDIT3 with high AUC values. It was reported that OA was induced due to the activation of subchondral osteoblasts during overstress.^64^ *In vitro*, stretching osteoblasts and submitting them under fluid shear stress can be used as the experimental models of OA to study the effect of mechanical stress on subchondral bone.^65^ Thus, we induced osteoblast progenitor cells differentiate into osteoblasts *in vitro*, and these autophagy genes were detected to be highly expressed after over mechanical loading *in vitro*. Next, we used DMM model and 24-month-old mice to verify the expression of the ARGs in knee subchondral bone. We found that in the DMM model, at 2 weeks after operation, that is, at the early stage of OA,^14^^,^^66^ our autophagy genes were activated. And with the progress of DMM, the expression of these genes increased, which may be related to the function of osteoblasts increased gradually during the occurrence and development of OA to regulate bone remodeling. Of course, ARGs in 24-month-old mice also showed upregulated situation. Moreover, we conducted analysis on human OA samples, specifically focusing on the ARGs in the subchondral bone tissue. Previous Imaging analysis of human samples has indicated that the medial tibial plateau exhibits thicker plate and lager bone volume compared to the lateral side. These findings suggest that bone formation is more pronounced in the medial tibial plateau than in the lateral tibial plateau, possibly indicating a more significant activation of osteoblasts in this region.^67^ Thus, when verifying the three key diagnostic genes, we selected the medial and lateral sides of the tibial plateau of the same patient as the OA group and the control group respectively. The results were consistent with the predicted results, which confirmed its reliability. These results suggest that autophagy of osteoblasts may play a key role in the pathological process of OA, and DDIT3, JUN, VEGFA all have potential to be used as a diagnostic marker and potential therapy for OA.

Recent researches have highlighted the crucial role of osteoblast activity in OA.^68^^,^^69^^,^^70^ Among them, osteoblasts are known for their ability to produce various growth factors including bone morphogenetic protein (BMPs), transforming growth factor β (TGF-β), and insulin-like growth factor (IGF) and so forth,^69^^,^^71^ which are essential for regulating the development of OA. Moreover, these growth factors contribute significantly to cartilage homeostasis.^71^^,^^72^ Additionally, under the influence of high fluid flow shear stress levels, the soluble factors secreted by osteoblasts lead to a reduction in the synthetic metabolism and an enhancement in the catabolic metabolism of chondrocytes.^73^ Our results also suggest that the secretion of osteogenic factors may be the key to regulating cartilage metabolism. *In vitro* co-culture experiments have provided insights into the molecular and cellular interactions between chondrocytes and osteoblasts.^69^ Specifically, studies have shown that co-culturing osteoblasts from sclerotic and non-sclerotic areas of OA patients with chondrocytes leads to accelerated differentiation and hypertrophy of chondrocytes by osteoblasts from sclerotic regions.^74^ Currently, our experimentation involving co-culturing osteoblasts with cartilage explants revealed that knocking down osteoblastic ARGs resulted in decreased osteoblast activity. Remarkably, this intervention was able to attenuate cartilage degradation induced by IL-1β stimulation. Collectively, these findings underscore the critical involvement of osteoblastic ARGs in OA progression. Therefore, targeting osteoblastic ARGs expression holds promise as an effective therapeutic strategy for managing OA.

### Limitations of the study

Despite these findings, the study still has some limitations. First, our bioinformatics analysis utilizes GEO database, and there is no high-throughput sequencing data for new clinical samples to verify the results of the current study. Second, this study does not collect the joint tissue from normal people, and there is no reasonable and correct way to collect samples of early OA. It can only be verified as a diagnostic marker in the early stage of animal DMM model but not in the OA patients. In addition, the upstream and downstream regulatory pathways of identified biomarkers need to be further studied.

## STAR★Methods

### Key resources table

**Table:** 

REAGENT or RESOURCE	SOURCE	IDENTIFIER
**Antibodies**		

Mouse monoclonal anti-osteocalcin	Santa Cruz	Cat#sc-365797 RRID:AB_10859392
Rabbit polyclonal anti-LC3	Proteintech	Cat#14600-1-AP RRID:AB_2137737
Rabbit polyclonal anti-CHOP (DDIT3)	Proteintech	Cat#15204-1-AP RRID:AB_2292610
Rabbit polyclonal anti-JUN	Proteintech	Cat#24909-1-AP RRID:AB_2860574
Rabbit polyclonal anti-VEGFA	Proteintech	Cat#19003-1-AP RRID:AB_2212657
Rabbit monoclonal anti-COL2	Servicebio	Cat#GB11021-100 RRID:AB_3097800
Rabbit polyclonal anti-MMP13	Proteintech	Cat#18165-1-AP
Rabbit monoclonal anti-COL1	Abcam	Cat#ab255809 RRID: AB_3097801
Rabbit monoclonal anti-RUNX2	Cell Signaling Technology	Cat#12556 RRID:AB_2732805
Rabbit monoclonal anti-Osteocalcin	ABclonal	Cat#A20800 RRID:AB_3097802
Mouse monoclonal anti-GAPDH	Proteintech	Cat#60004-1-Ig RRID:AB_2107436

**Deposited data**		

GSE147390	GEO	https://www.ncbi.nlm.nih.gov/geo/query/acc.cgi?acc=GSE147390
GSE51588	GEO	https://www.ncbi.nlm.nih.gov/geo/query/acc.cgi?acc=GSE51588
GSE114007	GEO	https://www.ncbi.nlm.nih.gov/geo/query/acc.cgi?acc=GSE114007
GSE82107	GEO	https://www.ncbi.nlm.nih.gov/geo/query/acc.cgi?acc=GSE82107

**Oligonucleotides**		

Si-DDIT3-1 Forward Primer: GGCUCAAGCAGGAAAUCGA Reverse Primer: UCGAUUUCCUGCUUGAGCC	TsingkeBiotechnologyCo.,Ltd.	N/A
Si-DDIT3-2 Forward Primer: GAUUCCAGUCAGAGUUCUA Reverse Primer: UAGAACUCUGACUGGAAUC	TsingkeBiotechnologyCo.,Ltd.	N/A
Si-JUN-1 Forward Primer: GUGCCUACGGCUACAGUAA Reverse Primer: UUACUGUAGCCGUAGGCAC	TsingkeBiotechnologyCo.,Ltd.	N/A
Si-JUN-2 Forward Primer: CAGUAACCCUAAGAUCCUAAA Reverse Primer: UUUAGGAUCUUAGGGUUACUG	TsingkeBiotechnologyCo.,Ltd.	N/A
Si-VEGFA-1 Forward Primer: GCAGCUUGAGUUAAACGAA Reverse Primer: UUCGUUUAACUCAAGCUGC	TsingkeBiotechnologyCo.,Ltd.	N/A
Si-VEGFA-2 Forward Primer: CCUUGUUCAGAGCGGAGAA Reverse Primer: UUCUCCGCUCUGAACAAGG	TsingkeBiotechnologyCo.,Ltd.	N/A

**Software and algorithms**		

GeneCards	N/A	http://www.genecards.org
MSigDB	N/A	https://www.gsea-msigdb.org/gsea/msigdb/
R version 4.1.1	N/A	https://www.r-project.org/
Graphpad Prism	N/A	https://www.graphpad.com/
limma	Bioconductor	https://bioconductor.org/packages/limma/
Metascape	N/A	https://metascape.org/
Cytoscape version 3.8.0	N/A	https://cytoscape.org
ImageJ	N/A	https://imagej.nih.gov/ij/

**Experimental models: Organisms/strains**		

C57BL/6J mice: DMM model	Glasson et al.^75^	N/A

### Resource availability

#### Lead contact

Further information and requests for resources and reagents should be directed to and will be fulfilled by the lead contact, Hang Fang (fanghang@smu.edu.cn).

#### Materials availability

This study did not generate new unique reagents.

#### Data and code availability

• This paper analyzes existing publicly available data. Accession numbers are listed in the key resources table. All data reported in this paper will be shared by the lead contact upon request.
• All original code has been included in the supplemental information.
• Any additional information required to reanalyze the data reported in this paper is available from the lead contact upon request.

### Experimental model and study participant details

#### Human tissue specimens

We recruited the tibial plateau of 10 patients who underwent joint replacement at the Third Affiliated Hospital of Southern Medical University, including cartilage and subchondral bone, excluding such as cancer, diabetes, Rheumatoid disease and other patients. All clinical samples used for scientific research were obtained with informed consent from all participants. This study was approved by the Ethics Committee of the Third Affiliated Hospital of Southern Medical University. The OA group (Late-OA) consisted of subchondral bone and cartilage from the medial tibial plateau of 10 OA patients who had undergone total knee replacement surgery. For the control group (Early-OA), subchondral bone and cartilage samples from the lateral tibial plateau, exhibiting less cartilage damage, were utilized. Detailed information about the participants is provided in Table S1.

#### Animals and OA model

All procedures and experiments were approved by the Animal Ethics Committee of the South China Agricultural University and complied with the guidelines of the Care and Use of Laboratory Animals. We purchased 8-week-old male and 4-month-old as well as 24-month-old C57BL/6 J mice from Experimental Animal Centre of Southern Medical University (Guangzhou, China). The destabilization of the medial meniscus (DMM)^75^ of the right knee joint of 8-week-old male mice was divided into OA group and the contralateral knee joint was regarded as the sham group. To explore the role of ARGs in DMM mouse model, eighteen 8-week-old male C57BL/6J mice were randomly divided into 3 groups to induce OA model. After DMM modeling, the mice (6 mice per time point) were sacrificed either 2, 4, or 8 weeks later. On the other hand, male mice ages 4 and 24 months were used to study the role of ARGs in age-associated spontaneous OA (5 mice/group).^76^ The study animals were housed in a specific-pathogen-free room with five mice per cage and provided with a consistent diet. Their living environment was maintained at a comfortable temperature and humidity, and their circadian rhythm was maintained at a 12-h cycle. Mice were euthanized by excessive ketamine/xylazine anesthetic injection, followed by cervical dislocation.

#### Cell culture

The skulls of C57 suckling mice born 2–3 days old were acquired, and then incubated with trypsin at 37°C for 25 min for digestion, and subsequently, the trypsin was removed. Following this, the tissues were cut up, and 1% type I collagenase was added to digest them at 37°C for 60 min. Upon completion of this digestion period, the tissues were centrifuged at 1000 rpm for 4 min, and the resulting supernatant was then discarded. The α-MEM with 10% FBS was added to mix tissues and evenly paved in the petri dish to obtain osteoblast progenitor cells, which were subsequently cultured in α-MEM with 10% FBS, 50 μg/mL ascorbic acid, and 10 mM β-glycerophosphate in a 5% CO2 humidified incubator at 37°C. In order to further study the mechanisms of the expression of key genes during over mechanical loading of osteoblasts, the osteoblasts were then cultured on the cell tension culture plate (cat#BF-3001C, Flexcell, USA). The FlexcellFX3000 stress stretching was carried out, and the stress program in the Flexcell software was set with a stress intensity of 20%, frequency of 0.5Hz, time duration of 24 or 48 h, and a waveform of 1/2sin.

### Method details

#### Data acquisition and preprocessing

A total of 435 cell markers associated with osteoarthritic osteoblasts were extracted from the single-cell RNA sequencing data of GSE147390. The 367 autophagy-related genes were obtained from the GeneCards public database (http://www.genecards.org). The training dataset (GSE51588) consisted of RNA sequencing (RNA-seq) data isolated from knee subchondral bone of 40 OA subjects and 10 healthy control subjects. The dataset was obtained from the Gene Expression Omnibus (GEO) database (https://www.ncbi.nlm.nih.gov/geo/). Subsequently, we retrieved two datasets for external validation. The GSE114007 dataset included whole-genome microarray profiling analyses from human knee joints that were site-matched. And the GSE82107 dataset included transcriptome data from knee joints obtained from 7 healthy individuals and 10 OA patients. To minimize divergence, we transformed the RNA-seq data into Transcripts-per-Million (TPM) format and then used the sva package in R software (version 3.6.3) to mitigate batch effects. Detailed information about the public datasets is provided in Table S2.

#### PPI identification

We utilized the STRING database to construct the protein-protein interaction (PPI) network, considering the minimum required interaction score of 0.4. whereafter, the Cytoscape software (version 3.8.0), a widely used tool, was employed to measure the significance of genes in the network. Then we calculated node scores via Cytohubba plug-in unit. The "Degree"," closeness " and " EPC " three of app algorithm were selected as core method for constructing PPI network. Consequently, we included the top 40 genes with the highest degree in further analysis.

#### Multiple machine learning approaches

To identify optimal autophagy-related biomarkers in osteoarthritis (OA), we utilized a combination of machine learning techniques^77^ to screen for key genes in the training dataset. Firstly, we employed the Boruta algorithm^78^ to conduct preliminary variable feature selection and identified the important genes from the dataset. The attributes marked as "Confirmed" were retained, whereas those labeled as "Tentative" or "Rejected" were discarded. The Extra Trees Classifier,^79^ a member of the Random Forest Classifier family, was then utilized in our analysis. This classifier employs multiple decision trees that are trained and aggregated for predictions. During the training process, a subset of features is randomly selected at each split in the trees, and their significance in relation to the final prediction is evaluated. By considering the "Accuracy" and "Impurity" scores for all markers, the Extra Trees Classifier generates a ranking of the most significant features in the training dataset. Finally, we performed Lasso regression using the glmnet package with 10-fold cross-validation to reduce the influence of irrelevant or noisy features on the potential model’s performance.

#### Construction of important feature model

The SVM method with recursive feature elimination (RFE)^80^ was utilized to construct the most effective features for Differentially Expressed Genes (DEGs). In addition, the Random Forest package in the R software was employed to construct the complete diagnosis model, with parameters set as ntree = 500 and mtry = 3. The Recursive Feature Elimination (RFE) algorithm is a backward selection technique, which begins with all the features and eliminates the weakest one until a specified number of features remains. Besides, the Random Forest algorithm measures the average decrease in impurity associated with each feature to assess its importance and constructs the diagnosis model accordingly. By combining these two techniques, either RFE or Random Forest ensures that the final feature set is both informative and applicable to new data.

#### Gene pathway annotation and analyses

The gene functional comprehensive set was performed using either the clusterProfile software in R or the Metascape website (https://metascape.org/). Both tools were employed to investigate and comprehend the biological implications of a long list of significant DEGs. We explored the gene ontologies (GO) pathway, Kyoto Encyclopedia of Genes and Genomes (KEGG) pathways, diseases and functions, as well as Wiki pathways associated with OA. Gene Set Enrichment Analysis (GSEA) and Gene set variation analysis (GSVA) was utilized to analyze the pathway patterns between the OA and Non-OA groups of samples, while different Normalized Enrichment Scores (NES) were used to represent the activity level of the pathways for each other. the defined gene symbols set “c5.go.v2023.2.Hs.symbols.gmt”, “c2.cp.kegg.v2023.2.Hs.symbols.gmt” and “c2.cp.wikipathways.v2023.2.Hs.symbols.gmt” was downloaded from the Molecular Signature Database. Terms with a statistical significance of adjusted *p* value <0.05 were deemed as significant.

#### Single-cell classification atlas

The raw single-cell data was obtained from the Gene Expression Omnibus (GEO) database under accession number GSE147390. The Seurat package (version 4.0.2) for the R programming language was employed to import and analyze the data. The inclusion criteria for barcodes were as follows:1. The feature of RNA should be more than 200 and less than 8000. 2.The proportion of mitochondrial genes should be less than 25%. 3.The proportion of erythrocyte genes should be less than 10%. We performed Principal components analysis (PCA) and used significant principal components (PCs) as input for graph-based clustering. For cluster identification, the “Find Clusters” function was utilized, which implements the shared nearest neighbor (SNN) modularity optimization-based clustering algorithm. 20 PCA components were used with resolutions ranging from 0.1 to 0.8, resulting in seven to sixteen. Subsequently, to further determine the optimal threshold, we screened the most stable overall clustering of 11 clusters with a resolution of 0.4 using osteoblast-specific cell markers. DEGs in each cell cluster were identified using the default parameters of the “Find All Markers” function from Seurat applied to the normalized gene expression data.

#### Cartilage explants

The tibial plateau cartilage explants were isolated from 3-week-old male C57 mice by removing the tibia below the growth plate and retaining the tibial plateau above it. Subsequently, under microscopic observation, the synovium and other soft tissues were carefully removed. The isolated explants were then placed in a 6-well plate and cultured in DMEM/F12 medium supplemented with 20% fetal bovine serum for a period of 3 days. Following the culture period, the vitality of the explants was assessed based on the appearance of the culture medium. Subsequently, the explants were co-cultured with osteoblasts as part of the experimental procedure.

#### SiRNA transfection

The ARGs mRNA suppression is achieved by using small interference RNA (siRNA). According to the product specification, 70 nM of DDIT3, JUN, VEGFA siRNA or siRNA negative control (NC) (Tsingke, Beijing, China) were transfected into preosteoblasts with lipofectamine 3000 (2.5 μ L/mL) for 24 h. Subsequently, the preosteoblasts were treated with α-MEM containing 10% FBS, 50 μg/mL ascorbic acid, and 10 mM β-glycerophosphate for 48 h (WB). The cells were performed with RIPA for western blot analysis to evaluate the effect of siRNA transfection.

#### Co-culture

The explants implanted in the supra-chamber of six-hole transwell plate (cat#3412, Corning, USA) were stimulated with 10 ng/ml IL-1 β and PBS (Blank), and then co-cultured with osteoblasts cultured with α-MEM containing 10% FBS, 50 μg/mL ascorbic acid, and 10 mM β-glycerophosphate in the subplate chamber of transwell. Osteoblasts transfected with siRNA were co-cultured with IL-1 β-stimulated explants. After a 3-day incubation period, the explants fixed with 4% paraformaldehyde were subjected to subsequent histological observation, while the osteoblast proteins collected in RIPA lysate underwent Western blot analysis for further investigation.

#### Histological observation

The knee samples were obtained from human knee joint specimens, OA mice and explants, followed by fixation in 4% paraformaldehyde for 2 days, and subsequent decalcification for 21 days. After this, the samples were dehydrated, embedded, and sliced. Routine safranine O-fast green staining and OARSI score analysis^81^ were utilized to assess histopathological variances among the groups. The Safranin O staining and grading of mouse tibial plateau cartilage explants were assessed following a previously established method.^82^ In addition, immunofluorescence (IF) staining was conducted to examine the expression of ARGs in the control group and the OA group. To be specific, the sections were dewaxed, had their antigens repaired, and were sealed with horse serum. In the next step, the primary antibodies of osteocalcin (OCN) (cat#sc-365797, Santa Cruz, USA) were incubated with primary antibodies of LC3 (cat#14600-1-AP, Proteintech, Wuhan, China), DDIT3 (cat#15204-1-AP, Proteintech, Wuhan, China), JUN (cat#24909-1-AP, Proteintech, Wuhan, China) and VEGFA (cat#19003-1-AP, Proteintech, Wuhan, China) to detect the co-expression and localization of ARGs and OCN. Secondary antibodies conjugated with fluorescent tags were added for immunofluorescent staining, followed by incubation at room temperature for 1 h in the dark. The last step was to seal the slides with DAPI (cat#F6057, Sigma-Aldrich, USA). To start the immunohistochemical staining (IHC) process, the sections were treated with 3% hydrogen peroxide for 10 min to eliminate endogenous peroxidase activity. Following this, sections were blocked with 1% goat serum (cat#AR0009, Boster, Wuhan, China) for 1 h at room temperature. Subsequently, the sections were incubated with the primary antibody COL2 (cat#GB11021-100, Servicebio, Wuhan, China) and MMP13(cat#18165-1-AP, Proteintech, Wuhan, China) at 4°C for 14 h. After antibody incubation, the sections need to be incubated with the species-matched horseradish peroxidase-conjugated secondary antibody (Jackson ImmunoResearch Laboratories, USA) at room temperature for 1 h. Finally, the color development can be achieved by applying 3,3'-diaminobenzidine (DAB) on the sections and then counterstain with hematoxylin. Finally, all sections were observed under an upright microscope (OLYMPUS, Japan).

#### Western blotting analysis

The first step in the experimental procedure involved using the RIPA lysis buffer (cat#FD009, Fudebio, Hangzhou, China), which contained phosphatase and protease inhibitors (cat#FD1002 and cat#FD1001, Fudebio, Hangzhou, China), to fully lyze cells and ground tissue samples on ice. Following this, the lysed sample was promptly centrifuged at 12000g, 4°C, resulting in the removal of the supernatant. Subsequently, the protein was quantified using Pierce^TM^ BCA Protein Assay Kits (cat#A55864, Thermo Scientific, USA), after which 5X loading buffer (cat#FD002, Fudebio, Hangzhou, China) was added and the mixture was heated in a 95°C metal bath for 5 min. Finally, the cell and tissue products, each with a protein amount of 30ug per group, were subjected to analysis by SDS-PAGE and subsequently transferred to a nitrocellulose membrane (Millipore, Billerica, MA, USA). Blots were probed with primary antibodies against JUN (cat#24909-1-AP, Proteintech, Wuhan, China), DDIT3 (cat#15204-1-AP, Proteintech, Wuhan, China), VEGFA (cat#19003-1-AP, Proteintech, Wuhan, China), LC3 (cat#14600-1-AP, Proteintech, Wuhan, China), Collagen 1a (COL1A1, cat#ab255809, Abcam, Cambridge, MA, USA), RUNX2 (cat#12556, Cell Signaling Technology, USA), Osteocalcin (OCN, cat#A20800, ABclonal, Wuhan, China), GAPDH (cat#60004-1-Ig, Proteintech, Wuhan, China), and immunoreactive proteins were revealed using an FDbio-Dura ECL Kit (cat#FD8020, Fudebio, Hangzhou, China). The evaluation of Western blot data involved quantifying the gray values of the western blot bands using ImageJ in both the control and experimental groups. To assess the protein level, the gray value of the protein of interest in each group was normalized by dividing it by the gray value of the internal reference protein. This method provided an estimate of the protein level in both the control and experimental groups.

### Quantification and statistical analysis

The statistical analyses for this study were conducted using R software (version 4.1.1) and GraphPad Prism 9 (version 9.3.0). Normalized expression profile data from GSE51588 was integrated with the clinical phenotype information of each sample in the dataset. This integrated data was then used to analyze the Seurat expression profile of the single-cell sequencing dataset GSE147390. Receiver operating characteristic (ROC) curves were generated, and the areas under the curve (AUCs) were calculated using the pROC package. For comparisons between two groups, either the Student’s t test or the Wilcoxon rank-sum test was employed. The Kruskal–Wallis test or one-way ANOVA was utilized for comparisons involving three or more groups. All statistical tests were two-tailed.

Statistical significance is denoted as follows: ∗*p* < 0.05; ∗∗*p* < 0.01; ∗∗∗*p* < 0.001; ∗∗∗∗*p* < 0.0001; ns, *p* > 0.05. All of the statistical details of experiments can be found in the figure legends.

## Supplemental information

Document S1. Figures S1–S4, Tables S1 and S2

Data S1. Original code

Data S2. The supplemental original images for western blot
