# Supplementary material for: Identification of osteoblastic autophagy-related genes for predicting diagnostic markers in osteoarthritis
Source: iScience. 2024 May 27;27(6):110130. doi: 10.1016/j.isci.2024.110130 (PMC11215306; doi:10.1016/j.isci.2024.110130)
Supplement: Document S1. Figures S1–S4, Tables S1 and S2 [file mmc1.pdf]

iScience, Volume *27*

Supplemental information

Identification of osteoblastic autophagy-related genes for predicting diagnostic markers in osteoarthritis

Rulong Cai, Qijun Jiang, Dongli Chen, Qi Feng, Xinzhi Liang, Zhaoming Ouyang, Weijian Liao, Rongkai Zhang, and Hang Fang

Supplemental Figures

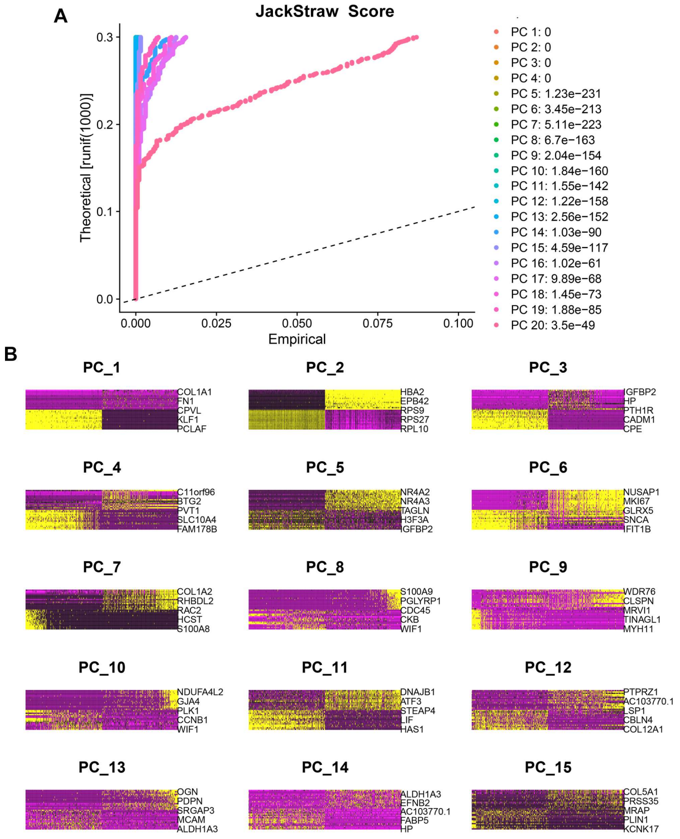

Figure S1 (Relates to Figure 2): PCA dimensionality Reduction Visualization for Single-Cell Dataset. A) The JackStraw score to evaluate the significance of each principal component obtained from PCA analysis. And identify which components represent true signals, facilitating further data interpretation and analysis. The principal component signals above the standard threshold were chosen for subsequent data interpretation and analysis. B) Heatmaps were used to display the specificity of upregulated and downregulated genes within each principal component, while also concurrently presenting their expression levels across different barcodes.

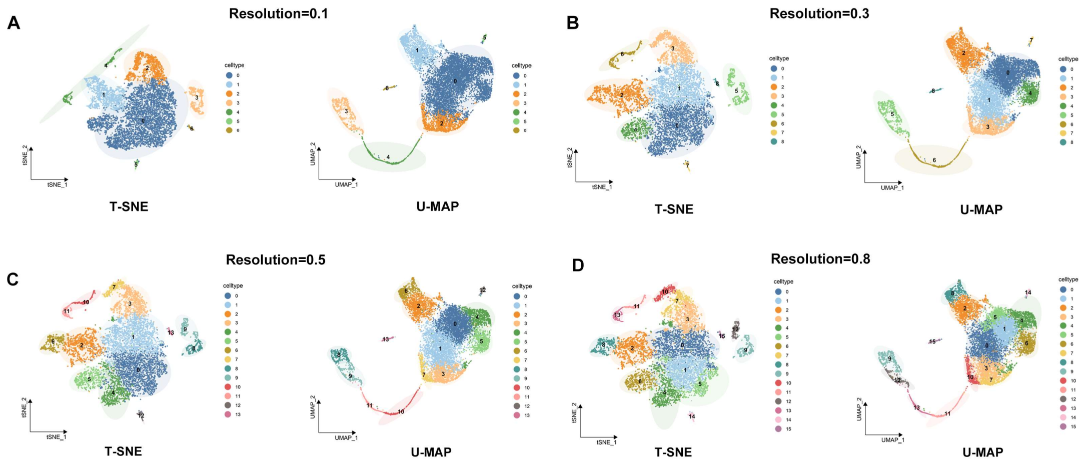

Figure S2 (Relates to Figure 2): Clustering strategy for different cell types in GSE147390. Visualizations of the t-SNE and UMAP dimensionality reduction were performed on different cell populations at resolutions of 0.1 A), 0.3 B), 0.5 C), and 0.8 D).

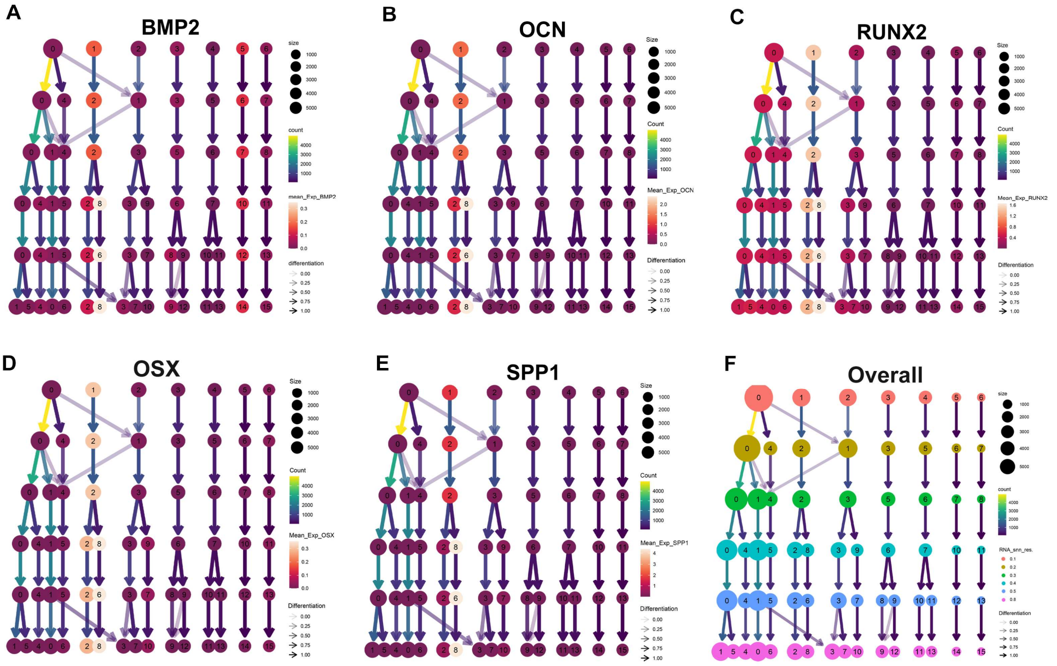

Figure S3 (Relates to Figure 2): The expression of the osteoblast-specific markers across diverse resolution. The expression of the osteoblast-specific markers A) BMP2, B) OCN, C) RUNX2, D) OSX and E) SPP1 primarily exhibit distinct distribution patterns across different groups categorized by dimensionality reduction. F) The comprehensive results.

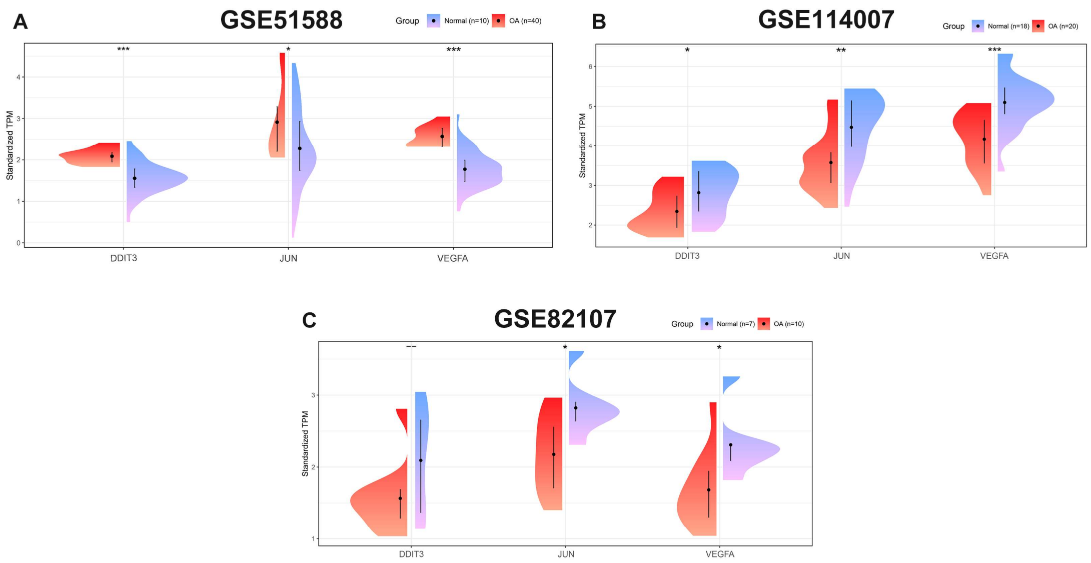

Figure S4 (Relates to Figure 5): The expression of the ARGs in each data set. DDIT3, JUN and VEGFA exhibited significant up-regulation in subchondral bone of OA patients A) (GSE51588), while these three markers were down-regulated in osteoarthritis cartilage B) (GSE114007) and C) synovium (GSE82107).

Supplemental tables

Table S1. Clinical parameters of all the participants used in this study. Related to STAR Methods.

Parameters	OA (n=10)
Gender	
Male	4(40%)
Female	6(60%)
Aged(average)	68.2
Ethnicity	Han Chinese

Table S2. Clinical parameters of all the bulk-RNA samples used in this study. Related to STAR Methods.

**Parameters**	**GSE51588**		**GSE114007**		**GSE82107**	
	Normal (n=10)	OA (n=40)	Normal (n=18)	OA (n=20)	Normal (n=7)	OA (n=10)
Gender						
Male	4(8.0%)	18(36.0%)	13(72.2%)	8(40.0%)	-	-
Female	6(12.0%)	22(44.0%)	5(27.8%)	12(60.0%)	-	-
Age(average)	38.4	69.6	36.6	66.2	-	-
BMI	-	25.33 ± 3.12	32.4 ± 8.0	30.7 ± 8.1	-	-
