# Supplementary material for: Identification of osteoblastic autophagy-related genes for predicting diagnostic markers in osteoarthritis
Source: iScience. 2024 May 27;27(6):110130. doi: 10.1016/j.isci.2024.110130 (PMC11215306; doi:10.1016/j.isci.2024.110130)
Supplement: Data S1. Original code [file mmc2.pdf]

```
###################################confusion matrix for training and testing##################################################


rt <- read.table("GEO.data")

# Data preprocessing
rt[rt < 0] <- 0
rt <- log2(rt + 1)
data <- normalizeBetweenArrays(rt)

# Predict and generate confusion matrix
predictions <- predict(model, newdata = test)
cm <- confusionMatrix(predictions, ref)
cm_d <- as.data.frame(cm$table)
cm_d$diag <- cm_d$Prediction == cm_d$Reference
cm_d$ref_freq <- cm_d$Freq * ifelse(is.na(cm_d$diag), -1, 1)
cm_d[cm_d == 0] <- NA
cm_d$Reference <- factor(cm_d$Reference, levels = rev(levels(as.factor(cm_d$Reference))))

library(ggplot2)
plt1 <- ggplot(data = cm_d, aes(x = Prediction, y = Reference, fill = ref_freq)) +
  geom_tile() +
  scale_fill_gradient2(low = "red4", high = "orchid4", midpoint = 0, na.value = "white") +
  geom_text(aes(label = Freq), color = "white", size = 7) +
  theme_minimal()

cm_st <- round(as.data.frame(cm$overall), 2)

library(gridExtra)
plt2 <- tableGrob(cm_st)

grid.arrange(plt1, plt2, nrow = 1, ncol = 2,
             top = textGrob("Confusion Matrix", gp = gpar(fontsize = 25, font = 1)))

#ROC Curve
rt=read.table(expFile, header=T, sep="\t", check.names=F, row.names=1)
y=gsub("(.*)\\_(.*)", "\\2", colnames(rt))
y=ifelse(y=="con", 0, 1)

geneRT=read.table(geneFile, header=F, sep="\t", check.names=F)

for(x in as.vector(geneRT[,1])){
```

```
    roc1=roc(y, as.numeric(rt[x,]))
    ci1=ci.auc(roc1, method="bootstrap")
    ciVec=as.numeric(ci1)
    pdf(file=paste0("ROC.",x,".pdf"), width=5, height=5)
    plot(roc1, print.auc=TRUE, col="red", legacy.axes=T, main=x)
    text(0.39, 0.43, paste0("95% CI: ",sprintf("%.03f",ciVec[1]),"-",sprintf("%.03f",ciVec[3])),
col="red")
    dev.off()
}

rt=read.table(expFile, header=T, sep="\t", check.names=F, row.names=1)
y=gsub("(.*)\\_(.*)", "\\2", colnames(rt))
y=ifelse(y=="con", 0, 1)

geneRT=read.table(geneFile, header=F, sep="\t", check.names=F)

for(x in as.vector(geneRT[,1])){

    roc1=roc(y, as.numeric(rt[x,]))
    ci1=ci.auc(roc1, method="bootstrap")
    ciVec=as.numeric(ci1)
    pdf(file=paste0("ROC.",x,".pdf"), width=5, height=5)
    plot(roc1, print.auc=TRUE, col="red", legacy.axes=T, main=x)
    text(0.39, 0.43, paste0("95% CI: ",sprintf("%.03f",ciVec[1]),"-",sprintf("%.03f",ciVec[3])),
col="red")
    dev.off()
}


##############################################################################GEO
RNA-Seq analysis######################################################


#prepare data and DEGs identification
exprs_data <- cbind(exprs_group1, exprs_group2)

design <- model.matrix(~ group)

contrast.matrix <- makeContrasts(group2-group1, levels=design)
colnames(contrast.matrix) <- c('group2-group1')

fit <- lmFit(exprs_data, design)
fit <- contrasts.fit(fit, contrast.matrix)
fit <- eBayes(fit)
```

```
topGenes <- topTable(fit, coef='group2-group1', number=Inf)
fit = na.omit(fit)

k1 = (fit$P.Value < 0.05)&(fit$logFC < -logFC_cutoff)
k2 = (fit$P.Value < 0.05)&(fit$logFC > logFC_cutoff)
fit$change = ifelse(k1,"DOWN",ifelse(k2,"UP","NOT"))

write.table(fit,file = "limma.txt",sep="\t",quote=F)

DEG.limma <- fit

sameGene=intersect(row.names(DEG.limma),OS.Markers,autophagy)
geoOut=geo[sameGene,]


#enrich pathway analysis
genes=unique(as.vector(sameGene))

df <- bitr(genes,
           fromType = "SYMBOL",
           toType ="ENTREZID",
           OrgDb = org.Hs.eg.db)

n <- sort(fit$logFC, decreasing = T)
geneList <- n
names(n) <- df$ENTREZID

ego <- enrichGO(gene          = names(n),

                OrgDb         = org.Hs.eg.db,
                ont           = "ALL",
                pAdjustMethod = "BH",
                pvalueCutoff  = 0.05,
                qvalueCutoff  = 0.05,
                readable      = TRUE)
t(ego@result[1,])
g=ego@result
ego2 <- gseGO(geneList     = n,
              OrgDb        = org.Hs.eg.db,
              ont          = "ALL",
              minGSSize    = 0,
              maxGSSize    = 500,
              pvalueCutoff = 0.05,
```

```
                         verbose           = FALSE)
ego2 <- setReadable(ego2, OrgDb = org.Hs.eg.db, keyType = "ENTREZID")
t(ego2@result[1,])
a=ego2@result
kk <- enrichKEGG(gene               = df[,1],
                              organism          = 'hsa',
                              pvalueCutoff = 0.05)
kk <- setReadable(kk, OrgDb = org.Hs.eg.db, keyType = "ENTREZID")
a=kk@result
t(kk@result[1,])
kk2 <- gseKEGG(geneList          = n,
                          organism          = 'hsa',
                          minGSSize        = 0,
                          pvalueCutoff = 0.05,
                          verbose           = FALSE)
kk2 <- setReadable(kk2, OrgDb = org.Hs.eg.db, keyType = "ENTREZID")
a=kk2@result
t(kk2@result[1,])
pathview(gene.data    = geneList,
                pathway.id = "hsa04142",
                species        = "hsa",
                limit            = list(gene=max(abs(geneList)), cpd=1))

wp = enrichWP(gene, organism = "Homo sapiens")
wp <- setReadable(wp, OrgDb = org.Hs.eg.db, keyType = "ENTREZID")
t(wp@result[1,])
wp2 = gseWP(geneList, organism = "Homo sapiens")
wp2 <- setReadable(wp2, OrgDb = org.Hs.eg.db, keyType = "ENTREZID")
t(wp2@result[1,])

rp <- enrichPathway(gene, pvalueCutoff = 0.05, readable=TRUE)
a=rp@result
t(rp@result[1,])

rp2 <- gsePathway(geneList,
                                pvalueCutoff = 0.05,
                                pAdjustMethod = "BH")
rp2 <- setReadable(rp2, OrgDb = org.Hs.eg.db, keyType = "ENTREZID")
a=rp2@result
t(rp2[1,])


library(GOplot)
terms = data.frame( category = data$ONTOLOGY,
```

```
                                ID = data$ID,
                                term = data$Description,
                                genes = gsub("/", ",", data$geneID),
                                adj_pval = data$p.adjust)
genelist=as.data.frame(n)
genelist <- data.frame(ID = df$SYMBOL, logFC = genelist$n) #从已有“数据”中提取 genelist，1 列 ID，1 列 logFC。
circ <- circle_dat(terms, genelist)

edit(circle_dat)
edit(GOCircle)
GOCircle(circ)
pdf(file="circo2.pdf",width = 10,height = 10)
GOCircle(df,rad1=2, #内环半径
        rad2=3, #外环半径
        label.size= 3, #标签大小
        label.fontface = 'bold', #标签字体
        nsub=12, #显示的terms数，前10个。（画图前需要先把数据整理好，想要哪些term）
        zsc.col = c('#ad002aff', 'white', "#00468bff"), # z-score 颜色
        lfc.col = c('#cc0066')) #  基因 up 或 down 的颜色
dev.off()

############################################################Modeling methods for selecting important features##############################################

#Data preprocessing
data <- read.table(inputFile, header=TRUE, sep="\t", check.names=FALSE, row.names=1)

x <- as.matrix(data)
y <- gsub("(.*)_(.*)", "\\2", row.names(data))

#LASSO
library(glmnet)
fit <- cv.glmnet(x, as.factor(y), family = "binomial", alpha = 1, type.measure = 'deviance', nfolds = 10)

coef <- coef(fit, s = fit$lambda.min)
lassoGene <- row.names(coef)[-1]

write.table(lassoGene, file = "LASSO.gene.txt", sep = "\t", quote = FALSE, row.names = FALSE, col.names = FALSE)


#SVM-RF
```

```
library(caret)

rfeFuncs <- list(rf = rfFuncs, treebag = treebagFuncs, lda = ldaFuncs, lr = lrFuncs, nb = nbFuncs, lm
= lmFuncs, svmRadial = caretFuncs)


profiles <- lapply(names(rfeFuncs), function(method) {
  set.seed(2)
  rfe(x, y, sizes = c(1:10, 15, 30), rfeControl = rfeControl(functions = rfeFuncs[[method]], method
= "cv"))
})

pdf(file = "SVM-RFE.pdf", width = 6, height = 5.5)
par(las = 1)
for (i in seq_along(profiles)) {
  results <- profiles[[i]]$results
  x <- results$Variables
  y <- results$RMSE
  plot(x, y, xlab = "Variables", ylab = "RMSE (Cross-Validation)", col = "darkgreen", main =
names(profiles)[i])
  wmin <- which.min(y)
  wmin.x <- x[wmin]
  wmin.y <- y[wmin]
  points(wmin.x, wmin.y, col = "blue", pch = 16)
  text(wmin.x, wmin.y, paste0('N=', wmin.x), pos = 2, col = 2)
}
dev.off()

test <- data[, c("DDIT3", "VEGFA", "JUN")]
model <- train(y ~ ., data = test, method = "rf")
predictions <- predict(model, newdata = test)


library(Boruta)

features <- as.matrix(data[, -1])
target <- data$target

boruta_obj <- Boruta(x, y,doTrace = 2)
boruta_res <- run(boruta_obj)
print(boruta_obj)

important_features <- getSelectedAttributes(boruta_obj)
borutagene<-as.data.frame(important_features)
```

```
#EXTRA Classifier
if (!require(extraTrees)) {
  install.packages("extraTrees")
  library(extraTrees)
}

X <- data[, -ncol(data)]
y <- data[, ncol(data)]

model <- extraTrees(X, y,ntree=500,
                    nodeSize=1,family="bernoulli")

extraTreesqGene <- importance(model)

geneList[["LASSO"]]<-lassoGene
geneList[["SVM-RFE"]]<-SVMqGene
geneList[["boruta"]]<-borutagene
geneList[["extraTrees"]]<-extraTreesqGene

mycol<-c("any color")
pdf(file="venn.pdf", width=5, height=5)
venn(geneList,col<-mycol[1:length(geneList)],zcolor<-mycol[1:length(geneList)],box<-F,ilabels<-F)
dev.off()

intersectGenes<-Reduce(intersect,geneList)
write.table(file<-outFile, intersectGenes, sep<-"\t", quote<-F, col.names<-F, row.names<-F)

train<-read.table("data51588.txt",sep<-"\t",header<-T,check.names<-F,row.names <-1)
test_data1<-read.table("data82107.txt",sep<-"\t",header<-T,check.names<-F,row.names <-1)
test_data2<-read.table("data114007.txt",sep<-"\t",header<-T,check.names<-F,row.names <-1)

process_data <- function(rt) {
  dimnames <- list(rownames(rt), colnames(rt))
  data <- matrix(as.numeric(as.matrix(rt)), nrow = nrow(rt), dimnames = dimnames)
  t(avereps(data))
}

data1 <- process_data(train)
data2 <- process_data(test_data1)
data3 <- process_data(test_data2)

data1<-data1[intersectGenes,]
data2<-data2[intersectGenes,]
```

```
data3<-data3[intersectGenes,]
data<-cbind(data1,data2,data3)

batchType<-c(rep(1,ncol(data1)),rep(2,ncol(data2)),rep(2,ncol(data3)))
outTab<-ComBat(data, batchType,par.prior=TRUE)

train.data<-t(outTab[,colnames(data1)])
test.data1<-t(outTab[,colnames(data2)])
test.data2<-t(outTab[,colnames(data3)])

train.data<-as.data.frame(train.data)
model.rf <- randomForest(as.factor(train.data$type) ~ ., data = train.data, ntree = 100, mtry = 3)

var_importance <- importance(model)
write.table(var_importance,"vimp.xls",sep = "\t")

predictions <- predict(model,train.data)
print(predictions)

true_positive <- sum(predictions == "positive" & test_data$target_column == "positive")
false_positive <- sum(predictions == "positive" & test_data$target_column == "negative")

true_positive_rate <- true_positive / sum(test_data$target_column == "positive")
false_positive_rate <- false_positive / sum(test_data$target_column == "negative")


##########################################################single-cell
identification#############################################################


library(Seurat)
library(cowplot)

# Load the data and create initial Seurat object
rt <- read.table(inputFile, header = TRUE, sep = "\t", check.names = FALSE)
rt <- as.matrix(rt)
rownames(rt) <- rt[, 1]
exp <- rt[, -(1)]
dimnames <- list(rownames(exp), colnames(exp))
data <- matrix(as.numeric(as.matrix(exp)), nrow = nrow(exp), dimnames = dimnames)
data <- avereps(data)
pbmc <- CreateSeuratObject(counts = data, project = "seurat", min.cells = 3, min.features = 50,
names.delim = "_")
```

```
# Preprocess data
pbmc[["percent.mt"]] <- PercentageFeatureSet(object = pbmc, pattern = "^MT-")
pdf(file = "01.featureViolin.pdf", width = 10, height = 6)
VlnPlot(object = pbmc, features = c("nFeature_RNA", "nCount_RNA", "percent.mt"), ncol = 3)
dev.off()
pbmc <- subset(x = pbmc, subset = nFeature_RNA > 50 & percent.mt < 5)
pdf(file = "01.featureCor.pdf", width = 10, height = 6)
plot1 <- FeatureScatter(object = pbmc, feature1 = "nCount_RNA", feature2 = "percent.mt", pt.size
= 1.5)
plot2 <- FeatureScatter(object = pbmc, feature1 = "nCount_RNA", feature2 = "nFeature_RNA",
pt.size = 1.5)
CombinePlots(plots = list(plot1, plot2))
dev.off()
pbmc <- NormalizeData(object = pbmc, normalization.method = "LogNormalize", scale.factor =
10000)
pbmc <- FindVariableFeatures(object = pbmc, selection.method = "vst", nfeatures = 1500)
top10 <- head(x = VariableFeatures(object = pbmc), 10)
pdf(file = "01.featureVar.pdf", width = 10, height = 6)
plot1 <- VariableFeaturePlot(object = pbmc)
plot2 <- LabelPoints(plot = plot1, points = top10, repel = TRUE)
CombinePlots(plots = list(plot1, plot2))
dev.off()

# Perform PCA analysis
pbmc <- ScaleData(pbmc)
pbmc <- RunPCA(object = pbmc, npcs = 10, pc.genes = VariableFeatures(object = pbmc))

# Visualization of PCA results
pdf(file = "02.pcaGene.pdf", width = 10, height = 8)
VizDimLoadings(object = pbmc, dims = 1:4, reduction = "pca", nfeatures = 20)
dev.off()

pdf(file = "02.PCA.pdf", width = 6.5, height = 6)
DimPlot(object = pbmc, reduction = "pca")
dev.off()

pdf(file = "02.pcaHeatmap.pdf", width = 10, height = 8)
DimHeatmap(object = pbmc, dims = 1:4, cells = 500, balanced = TRUE, nfeatures = 30, ncol = 2)
dev.off()

pbmc <- JackStraw(object = pbmc, num.replicate = 100)
pbmc <- ScoreJackStraw(object = pbmc, dims = 1:10)
pdf(file = "02.pcaJackStraw.pdf", width = 8, height = 6)
JackStrawPlot(object = pbmc, dims = 1:10)
```

```
dev.off()


cluster_analysis_vis <- function(pbmc_data) {
  # Clustree visualization
  clustree(pbmc_data, node_colour = "SPP1", node_colour_aggr = "mean",
           node_size_range = c(6, 10), node_text_size = 3,
           edge_width = 2) +
    scale_colour_viridis(option = "rocket", begin = 0.3) +
    guides(size = FALSE,
           colour = guide_colourbar(title = "x",
                                    barwidth = 2,
                                    barheight = 30,
                                    order = 2),
           edge_colour = FALSE,
           edge_alpha = FALSE) +
    theme(legend.title = element_text(size = 20),
          legend.text = element_text(size = 15))

  # Find clusters
  resolution <- c(0.1, 0.2, 0.3, 0.4, 0.5, 0.8)
  pbmc_data <- FindNeighbors(pbmc_data, dims = 1:20)
  pbmc_data <- FindClusters(object = pbmc_data, resolution = resolution, verbose = TRUE)

  # Apply clustering results
  clustree(pbmc_data@meta.data, prefix = "RNA_snn_res.")
}

# Call the function with your pbmc data as input
cluster_analysis_vis(pbmc)



plot_umap_tsne <- function(pbmc_data) {
  rna_snn_list <- grep("^RNA_snn_res", colnames(pbmc_data@meta.data), value = TRUE)

  output_folder <- "UMAP-tSNE2"
  if (!dir.exists(output_folder)) dir.create(output_folder, showWarnings = FALSE)

  for (rna_snn in rna_snn_list) {
    tsne <- DimPlot(pbmc_data, reduction = "tsne", group.by = rna_snn, label = TRUE, label.size
= 8)
    umap <- DimPlot(pbmc_data, reduction = "umap", group.by = rna_snn, label = TRUE,
label.size = 8)
```

```
        p <- tsne + umap
        filename <- file.path(output_folder, paste0("UMAP_tSNE_", rna_snn, "_plot.png"))
        ggsave(filename, plot = p)
    }

    pbmc_data <- AddMetaData(pbmc_data, pbmc_data@reductions$umap@cell.embeddings,
col.name = c("UMAP_1", "UMAP_2"))
    pbmc_data <- AddMetaData(pbmc_data, pbmc_data@reductions$pca@cell.embeddings,
col.name = colnames(pbmc_data@reductions$pca@cell.embeddings))

    clustree_overlay(pbmc_data, prefix = "RNA_snn_res.", x_value = "UMAP_1", y_value =
"UMAP_2",
                                use_colour = "points", alt_colour = "blue")
}

plot_umap_tsne(pbmc)


#monocle
monocle_cds <- newCellDataSet(
    assays = list(counts = as.matrix(pbmc1@assays$RNA@counts)),
    rowData = DataFrame(gene_short_name = row.names(pbmc1@assays$RNA@counts)),
    colData = pd,
    featureData = fd,
    lowerDetectionLimit = 0.5,
    expressionFamily = negbinomial.size()
)

monocle_cds <- estimateSizeFactors(monocle_cds)
monocle_cds <- estimateDispersions(monocle_cds)
cds <- detectGenes(cds, min_expr = 0.1)

disp_table <- dispersionTable(monocle_cds)
disp.genes <- subset(disp_table, mean_expression >= 0.1 & dispersion_empirical >= 1 *
dispersion_fit)$gene_id
monocle_cds <- setOrderingFilter(monocle_cds, disp.genes)

pdf("train.ordergenes.pdf")
plot_ordering_genes(monocle_cds)
dev.off()

monocle_cds <- reduceDimension(monocle_cds, max_components = 2, reduction_method =
'DDRTree')
```

```
monocle_cds <- orderCells(monocle_cds)
orderCells <- function(cds,
                       root_state=NULL,
                       num_paths = NULL,
                       reverse=NULL){

  if(class(cds)[1] != "CellDataSet") {
    stop("Error cds is not of type 'CellDataSet'")
  }

  if (is.null(cds@dim_reduce_type)){
    stop("Error: dimensionality not yet reduced. Please call reduceDimension() before calling
this function.")
  }
  # reducedDimA, S, and K are not NULL in the cds
  if (any(c(length(cds@reducedDimS) == 0, length(cds@reducedDimK) == 0))) {
    stop("Error: dimension reduction didn't prodvide correct results. Please check your
reduceDimension() step and ensure correct dimension reduction are performed before calling
this function.")
  }

  root_cell <- select_root_cell(cds, root_state, reverse)

  cds@auxOrderingData <- new.env( hash=TRUE )
  if (cds@dim_reduce_type == "DDRTree"){
    if (is.null(num_paths) == FALSE){
      message("Warning: num_paths only valid for method 'ICA' in reduceDimension()")
    }
    cc_ordering <- extract_ddrtree_ordering(cds, root_cell)

    pData(cds)$Pseudotime <-   cc_ordering[row.names(pData(cds)),]$pseudo_time

    K_old <- reducedDimK(cds)
    old_dp <- cellPairwiseDistances(cds)
    old_mst <- minSpanningTree(cds)
    old_A <- reducedDimA(cds)
    old_W <- reducedDimW(cds)

    cds <- project2MST(cds, project_point_to_line_segment) #project_point_to_line_segment
can be changed into other states
    minSpanningTree(cds)                                                              <-
cds@auxOrderingData[[cds@dim_reduce_type]]$pr_graph_cell_proj_tree

    root_cell_idx <- which(V(old_mst)$name == root_cell, arr.ind=T)
```

```
    cells_mapped_to_graph_root <- which(cds@auxOrderingData[["DDRTree"]]$pr_graph_cell_proj_closest_vertex == root_cell_idx)
    if(length(cells_mapped_to_graph_root) == 0) { #avoid the issue of multiple cells projected to the same point on the principal graph
      cells_mapped_to_graph_root <- root_cell_idx
    }

    cells_mapped_to_graph_root <- V(minSpanningTree(cds))[cells_mapped_to_graph_root]$name

    tip_leaves <- names(which(degree(minSpanningTree(cds)) == 1))
    root_cell <- cells_mapped_to_graph_root[cells_mapped_to_graph_root %in% tip_leaves][1]
    if(is.na(root_cell)) {
      root_cell <- select_root_cell(cds, root_state, reverse)
    }

    cds@auxOrderingData[[cds@dim_reduce_type]]$root_cell <- root_cell

    cc_ordering_new_pseudotime <- extract_ddrtree_ordering(cds, root_cell) #re-calculate the pseudotime again

    pData(cds)$Pseudotime <- cc_ordering_new_pseudotime[row.names(pData(cds)),]$pseudo_time
    if (is.null(root_state) == TRUE) {
      closest_vertex <- cds@auxOrderingData[["DDRTree"]]$pr_graph_cell_proj_closest_vertex
      pData(cds)$State <- cc_ordering[closest_vertex[, 1],]$cell_state #assign the state to the states from the closet vertex
    }
    cds
  }
}

plot_cell_trajectory(monocle_cds, color_by = "State") +
  facet_wrap(~State, nrow = 1)

p1=plot_cell_trajectory(cds, color_by = "cell_type")    + scale_color_npg()
p2=plot_cell_trajectory(cds, color_by = "State")    + scale_color_nejm()
colour=c("#DC143C","#0000FF","#20B2AA","#FFA500","#9370DB","#98FB98","#F08080")
p3=plot_cell_trajectory(cds, color_by = "State")    + scale_color_manual(values = colour)
p1|p2|p3


p1 <- plot_cell_trajectory(monocle_cds, x = 1, y = 2, color_by = "cell_type2") +
  theme(legend.position='none',panel.border = element_blank()) +
```

```
    scale_color_manual(values = colour)
p2 <- plot_complex_cell_trajectory(monocle_cds, x = 1, y = 2,
                                   color_by = "cell_type2")+
    scale_color_manual(values = colour) +
    theme(legend.title = element_blank())
p1|p2

df <- pData(monocle_cds)



Time_diff <- differentialGeneTest(monocle_cds[ordergene,], cores = 1,
                                  fullModelFormulaStr = "~sm.ns(Pseudotime)")
Time_diff <- Time_diff[,c(5,2,3,4,1,6,7)]

Time_genes=as.character(Time_diff[,5])
Time_genes=as.character(Time_diff[,5])[389:419]
Time_genes <- Time_diff %>% pull(gene_short_name) %>% as.character()
plot_pseudotime_heatmap(monocle_cds[Time_genes,], num_clusters=4, show_rownames=T,
return_heatmap=T)

BEAM_res <- BEAM(monocle_cds[ordergene,], branch_point = 2, cores = 1)

BEAM_res <- BEAM_res[order(BEAM_res$qval),]
BEAM_res <- BEAM_res[,c("gene_short_name", "pval", "qval")]
```
