# Supplementary material for: Identification of osteoblastic autophagy-related genes for predicting diagnostic markers in osteoarthritis
Source: iScience. 2024 May 27;27(6):110130. doi: 10.1016/j.isci.2024.110130 (PMC11215306; doi:10.1016/j.isci.2024.110130)

Data S2

A) The original images for western blot of the subchondral bone from OA patients. Related to Figure 7H. Grouping from left to right is shown in Figure A.

B) The original images for western blot of 20% mechanical loading of osteoblasts. Related to Figure 5D. Grouping from left to right is shown in Figure B.

A

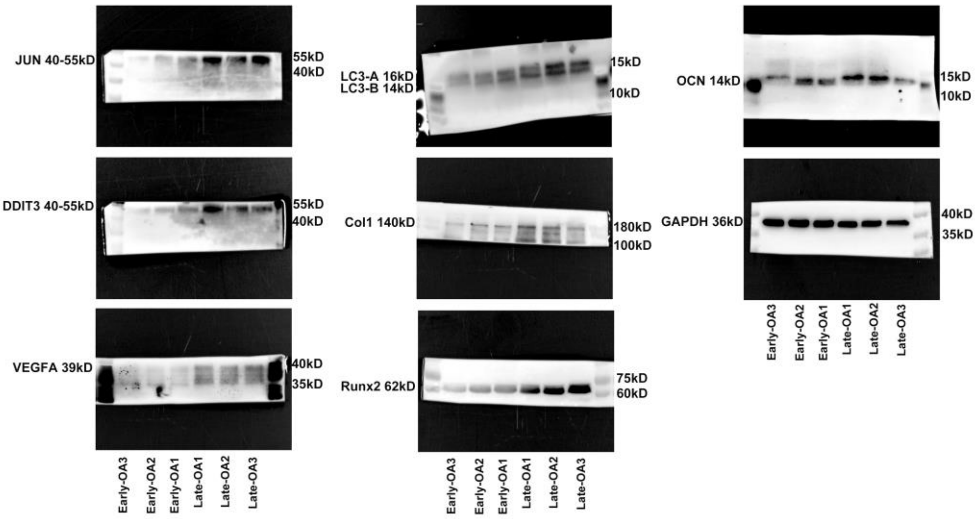

B

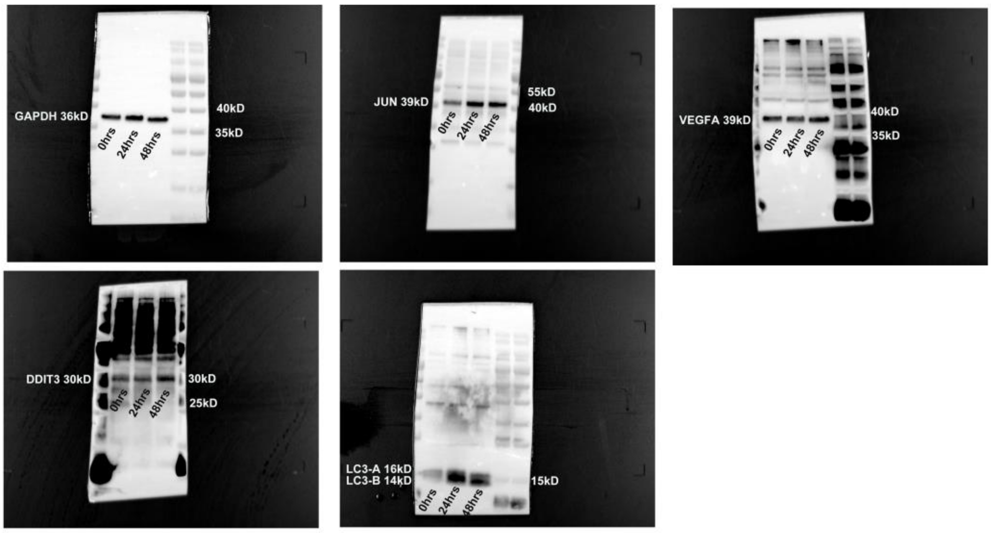

The original images for western blot of the osteoblasts transfected with siRNA. Related to Figure 8B.

Grouping from left to right is shown in Figure.

Grouping from top to bottom:

① Transfected with siDDIT3, ② Transfected with siJUN, ③ Transfected with siVEGFA

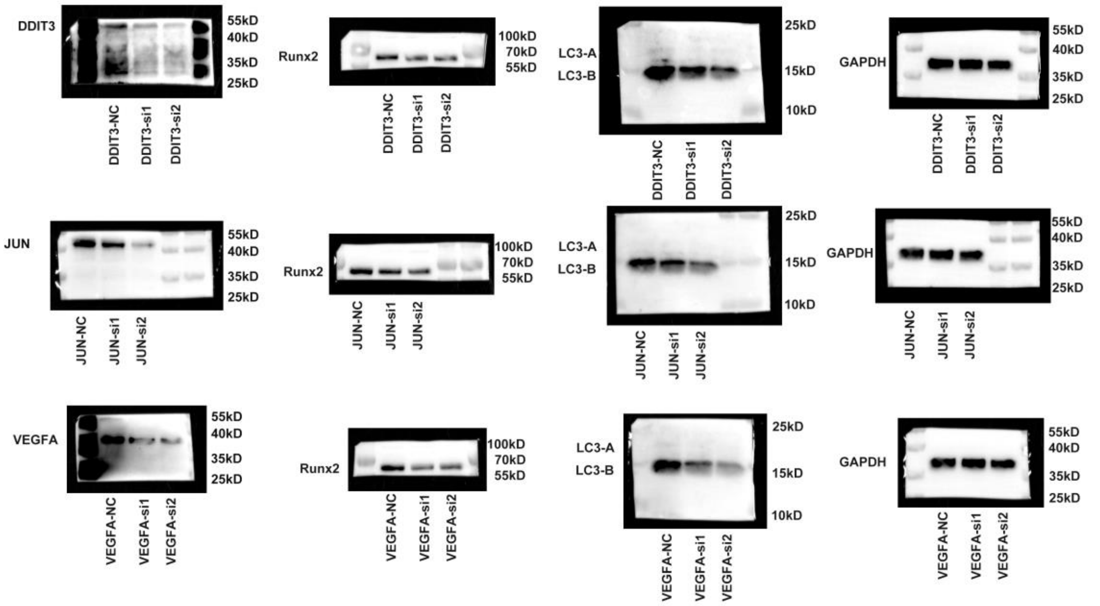

Supplement: Data S2. The supplemental original images for western blot [file mmc3.pdf]
